# Annexin A6 modulates TBC1D15/Rab7/StARD3 axis to control endosomal cholesterol export in NPC1 cells

**DOI:** 10.1007/s00018-019-03330-y

**Published:** 2019-10-29

**Authors:** Elsa Meneses-Salas, Ana García-Melero, Kristiina Kanerva, Patricia Blanco-Muñoz, Frederic Morales-Paytuvi, Júlia Bonjoch, Josefina Casas, Antonia Egert, Syed S. Beevi, Jaimy Jose, Vicenta Llorente-Cortés, Kerry-Anne Rye, Joerg Heeren, Albert Lu, Albert Pol, Francesc Tebar, Elina Ikonen, Thomas Grewal, Carlos Enrich, Carles Rentero

**Affiliations:** 1 Departament de Biomedicina, Unitat de Biologia Cel·lular, Facultat de Medicina i Ciències de la Salut, Universitat de Barcelona, 08036 Barcelona, Spain; 2 Centre de Recerca Biomèdica CELLEX, Institut d’Investigacions Biomèdiques August Pi i Sunyer (IDIBAPS), 08036 Barcelona, Spain; 3 Faculty of Medicine, Anatomy, University of Helsinki, 00014 Helsinki, Finland; 4 Minerva Foundation Institute for Medical Research, 00290 Helsinki, Finland; 5 Research Unit on BioActive Molecules (RUBAM), Department of Biological Chemistry, Institute for Advanced Chemistry of Catalonia (IQAC-CSIC), Barcelona, Spain; 6 School of Pharmacy, Faculty of Medicine and Health, University of Sydney, Sydney, NSW 2006 Australia; 7 Lipids and Cardiovascular Pathology Group, Biomedical Research Institute Sant Pau (IIB Sant Pau), Barcelona, Spain; 8 CIBERCV, Institute of Health Carlos III, Madrid, Spain; 9 Biomedical Research Institute of Barcelona-CSIC, Barcelona, Spain; 10 School of Medical Sciences, University of New South Wales, Sydney, NSW 2052 Australia; 11 Department of Biochemistry and Molecular Biology II: Molecular Cell Biology, University Medical Center Hamburg-Eppendorf, Martinistraße 52, 20246 Hamburg, Germany; 12 Department of Biochemistry, Stanford University School of Medicine, Stanford, USA; 13 Institució Catalana de Recerca i Estudis Avaçats (ICREA), 08010 Barcelona, Spain

**Keywords:** Cholesterol, Late endosomes, Rab7, NPC1, Annexin A6, Membrane contact sites

## Abstract

Cholesterol accumulation in late endosomes is a prevailing phenotype of Niemann-Pick type C1 (NPC1) mutant cells. Likewise, annexin A6 (AnxA6) overexpression induces a phenotype reminiscent of NPC1 mutant cells. Here, we demonstrate that this cellular cholesterol imbalance is due to AnxA6 promoting Rab7 inactivation via TBC1D15, a Rab7-GAP. In NPC1 mutant cells, AnxA6 depletion and eventual Rab7 activation was associated with peripheral distribution and increased mobility of late endosomes. This was accompanied by an enhanced lipid accumulation in lipid droplets in an acyl-CoA:cholesterol acyltransferase (ACAT)-dependent manner. Moreover, in AnxA6-deficient NPC1 mutant cells, Rab7-mediated rescue of late endosome-cholesterol export required the StAR-related lipid transfer domain-3 (StARD3) protein. Electron microscopy revealed a significant increase of membrane contact sites (MCS) between late endosomes and ER in NPC1 mutant cells lacking AnxA6, suggesting late endosome-cholesterol transfer to the ER via Rab7 and StARD3-dependent MCS formation. This study identifies AnxA6 as a novel gatekeeper that controls cellular distribution of late endosome-cholesterol via regulation of a Rab7-GAP and MCS formation.

**Electronic supplementary material:**

The online version of this article (10.1007/s00018-019-03330-y) contains supplementary material, which is available to authorized users.

## Introduction

The transmembrane NPC1 protein is essential for the efficient export of cholesterol from endolysosomes. Several other cytoplasmic players also contribute, via vesicular and/or non-vesicular pathways, to the exit of cholesterol from this compartment [1–3], including members of the oxysterol-binding protein (OSBP) family, such as oxysterol-related protein 1L (ORP1L), the small GTPases Rab7, Rab8 and Rab9, as well as the late endosome, membrane-anchored StARD3 and StARD3 N-terminal like (StARD3NL) proteins [3–6]. Despite the ability of these multiple players to contribute to cholesterol homeostasis, loss-of-function mutations in the NPC1 protein are dominant. Some of the cytoplasmic proteins that bind and transport cholesterol are also engaged in the formation and functions of membrane contact sites (MCS) that are emerging as important non-vesicular transfer mediators for lipids, cholesterol or calcium (Ca^2+^) between compartments [7–20].

StARD3 and Rab7 are critical for the regulation of MCS formation as well as cholesterol transfer between late endosomes and the endoplasmic reticulum (ER) [21–23]. StARD3 is ubiquitously expressed and anchored to the membrane of a late endosome subpopulation [24, 25], where it binds to the ER-resident vesicle-associated membrane protein-associated protein A (VAP-A) protein [26, 27]. Like other members of the START family, StARD3 could facilitate transport of cholesterol between late endosomes and other compartments such as the ER, mitochondria or plasma membrane [28–30]. However, despite its participation in cholesterol transfer between compartments via MCS, StARD3 overexpression did not increase cholesterol esterification via acyl-CoA:cholesterol acyltransferase (ACAT) in the ER [31, 32] and was unable to rescue late endosome-cholesterol accumulation in NPC1 mutant cells [23, 24].

At the same interface that connects late endosomes and ER compartments, the GTPase Rab7 regulates membrane trafficking, cholesterol homeostasis and contributes, together with protrudin and FYVE and coiled-coil domain containing 1 (FYCO1), in MCS dynamics [9–12]. In addition, Rab7 is responsible for endocytic transport between early endosomes, late endosomes, lysosomes, phago- and autolysosomes [3, 33, 34]. Within these compartments, Rab7 contributes to late endosome motility [35], cholesterol egress [36, 37], as well as early endosome maturation. While Rab7-GTP levels appear downregulated in cholesterol overloaded endosomes of NPC1 mutant cells, ectopic expression of wild type, constitutively active Rab7, or the adenoviral protein RIDα, can bypass (at least in part), the NPC1 defect to reduce late endosome-cholesterol accumulation [36, 38]. Given the complexity of these observations, we reasoned that yet unknown tethers or scaffolding proteins could control the dynamics of late endosome MCS, raising the possibility that yet unidentified player(s) or “gatekeepers” may fine-tune alternative late endosome-cholesterol transport routes in concert with NPC1. In fact, a recent publication identifies Gramd1b, an ER-sterol transport protein, interacting with NPC1 and transferring cholesterol from LE to the ER [39].

AnxA6, the largest member of the annexin family, has been implicated in the regulation of endo- and exocytic pathways, cholesterol homeostasis and the formation of multifactorial signaling complexes [40–42]. Like other annexins, the majority of AnxA6 binds to membranes in a Ca^2+^-dependent manner, yet cholesterol loading of late endosomes, using the NPC1 inhibitor U18666A or low-density lipoproteins (LDL), led to the recruitment of significant amounts of AnxA6 to the surfaces of late endosomes [43, 44]. AnxA6 was also enriched in late endosomes lacking functional NPC1 [45]. Moreover, AnxA6 overexpression led to the accumulation of cholesterol in late endosomes. Although these studies link AnxA6 with cholesterol export from endolysosomes [46], the underlying molecular mechanisms remain unclear. Here, we show that AnxA6 depletion alleviates the NPC1 mutant phenotype through two critical mechanisms: it triggers endogenous Rab7 activation by sequestering the Rab7-GTPase activating protein, TBC1D15; it also enables StARD3 to facilitate the function of MCS between late endosomes and the ER, aiding cholesterol export from endolysosomes. Our findings implicate AnxA6 inhibition as a novel strategy to rescue late endosome-cholesterol accumulation and identify the AnxA6/TBC1D15 complex as a potential therapeutic target for NPC disease.

## Materials and methods

### Materials

For primary and secondary antibodies, recombinant DNA, siRNA, chemicals and commercial assays, see Supplementary Table 1. Low-density lipoproteins (LDL, density 1.025–1.05 g/ml) were isolated from the plasma of normolipidemic volunteers by two sequential density gradient ultracentrifugation in KBr gradients [47]. Lipoprotein-deficient fetal calf serum (LPDS) was prepared by ultracentrifugation as described [48]. Before experiments, LDL and LPDS were dialyzed extensively against PBS and stored at 4 °C until use.

LDL protein concentration was determined by the bicinchoninic acid (BCA) method (Bio-Rad). Glutathione S-transferase (GST) and GST-fusion proteins (GST–AnxA6, Rab interacting lysosomal protein (RILP)-C33–GST, GST–perfringolysin O (PFO)) were produced in *E. coli* BL21 cells and purified using glutathione Sepharose 4B beads (GE Healthcare) as reported previously [49].

### Cell culture and transfections

Chinese hamster ovary wild type (CHO-WT), CHO-AnxA6 [43], CHO M12 and CHO 2-2 cells were grown in F12 (HAM) supplemented with 10% fetal bovine serum (FBS, Biological Industries), 2 mM l-glutamine (Sigma Aldrich), 100 units/ml penicillin (Biological Industries) and 100 μg/ml streptomycin (Biological Industries) at 37 °C, 5% CO_2_. CHO M12 and CHO 2-2 were kindly provided by Dr. L. Liscum (Tufts University School of Medicine, USA) and Dr. D. Ory (Washington University, USA), respectively. A431-WT, A431-A6 [50], mouse embryonic fibroblasts from wild type (MEF-WT) and AnxA6 KO-mice (MEF-A6ko) [51], COS-1 cells were cultured in DMEM supplemented with 10% (A431, COS-1) or 5% FBS (MEF), 2 mM l-glutamine (Sigma Aldrich), 100 units/ml penicillin (Biological Industries) and 100 μg/ml streptomycin (Biological Industries) at 37 °C, 5% CO_2_.

For transient transfections with fluorescently labeled AnxA6, TBC1D15 and Rab7 proteins, cells were incubated with GenJet Plus Reagent (SigmaGen Laboratories) following manufacturer’s instructions. For siRNA-mediated knockdown of AnxA6, TBC1D15 and StARD3, cells were transfected with 100 μM siRNA targeting mouse AnxA6, TBC1D15 and StARD3 (Santa Cruz) using Lipofectamine RNAiMax (Invitrogen) according to the manufacturer’s instructions. Studies were conducted 24 h (siTBC1D15) or 72 h (siAnxA6, siStARD3) after transfection. Scrambled siRNA served as negative control (Dharmacon).

### Generation of CHO M12-A6ko cells using the CRISPR/Cas9 system

For AnxA6 gene depletion in CHO M12 cells using CRISPR/Cas9 editing technology, guide RNAs targeting hamster AnxA6 were designed as described [52], and CHO M12 cells were transfected with pSpCas9(BB)-2A-Puro v2 (Addgene) carrying gRNAs against hamster AnxA6. 24 h after transfection, cells were selected for 48 h in puromycin (50 µg/ml). Clones were isolated by dilution and single clones were screened for AnxA6 gene knockout by western blotting and sequencing.

### Immunoblotting

Cells were lysed in lysis buffer (50 mM Tris–HCl, 150 mM NaCl, 1% Triton X-100, 0.1 mM CaCl_2_, pH 7.4) supplemented with protease/phosphatase inhibitors cocktail (1 mM Na_3_VO_4_, 10 mM NaF, 1 mM PMSF, 10 µg/ml leupeptin, 10 µg/ml aprotinin). Lysates were boiled in 1 × sample buffer, resolved on SDS-PAGE and transferred to nitrocellulose (Bio-Rad) or Immobilon-P (Millipore) membranes. Membranes were blocked in 5% non-fat milk, incubated overnight in primary antibodies, washed in TBST, incubated with HRP-conjugated secondary antibodies (Bio-Rad or Abcam, see Supplementary Table 1) and developed using enhanced chemiluminescence EZ-ECL (Biological Industries) and Fuji Medical X-ray films (Fujifilm). ImageJ software was used for quantitative analysis of WB bands [53].

### RNA extraction and quantitative real-time PCR

Total RNA was extracted using RNeasy Mini Kit (Qiagen) in accordance with the manufacturer’s protocol. 1 μg RNA was reverse-transcribed using High Capacity cDNA Reverse Transcription Kit (Applied Bioscience). In a final volume of 20 μl real-time PCR Brilliant SYBRGreen QPCR Master Mix (Agilent Technologies, Stratagene), 10 μl of 1:20 diluted cDNA was used as a template for PCR analysis using the LightCycler system (Roche Diagnostics), specific primers (see Supplementary Table 1) and standard PCR amplification protocol (10 min at 95 °C; 45 cycles of 30 s at 95 °C, 15 s at 60 °C and 30 s at 72 °C; and 10 s at 95 °C and 60 s at 65 °C) according to manufacturer’s instructions. Values were normalized to *Rpl13* gene in each sample.

### Preparation of liver homogenates

Mouse liver tissues were placed in Lysing Matrix tubes (MP Biomedicals) with homogenization buffer (10 mM Tris, 150 mM NaCl, 5 mM EDTA, pH 7.5) supplemented with protease/phosphatase inhibitors cocktail (see above). Samples were then homogenized in a FastPrep120 homogenizer (MP Biomedicals) and stored at − 20 °C. For immunoprecipitations (see below), liver homogenates were pre-cleaned with Protein A-agarose beads for 90 min at 4 °C before antibody incubation.

### Immunoprecipitation

Cells were grown on 10-mm dishes, washed with PBS and solubilized in lysis buffer (50 mM Tris, 150 mM NaCl, 1% Triton X-100, 0.1 mM CaCl_2_, pH 7.4), supplemented with protease/phosphatase inhibitors cocktail (see above). After centrifugation at 12,000*g* for 6 min at 4 °C, proteins from supernatants (200–400 µg) were incubated with 2 µg of rabbit polyclonal anti-AnxA6, rabbit polyclonal anti-TBC1D15 (Abcam) or rabbit IgG for 2 h at 4 °C, followed by 60 min with Protein A-agarose beads (Thermo Scientific). Immunoprecipitates were washed three times with lysis buffer and analyzed by western blotting.

### Pull-down assays

Cells were solubilized in pull-down buffer (50 mM Tris, 150 mM NaCl, 1% Triton X-100, 0.1 mM CaCl_2_, pH 7.3) supplemented with protease/phosphatase inhibitors cocktail (see above). Samples were centrifuged at 12,000*g* for 10 min at 4 °C. Proteins from post-nuclear supernatants (400–700 µg) were incubated with glutathione Sepharose 4B beads (GE Healthcare) coated with purified recombinant AnxA6–GST or RILP-C33–GST (40–70 µg) fusion protein for 2 h at 4 °C. GST was used as a negative control. Samples were washed three times, collected in 30 µL of 1× loading buffer and analyzed by western blotting.

### Subcellular fractionation

Late endosomes were isolated using sucrose gradients as described previously [43, 54]. Briefly, 25 × 10^6^ CHO-WT and CHO-A6 cells were used for each gradient. Cells were washed twice with cold PBS and collected. Cells were pelleted and resuspended in homogenization buffer (250 mM sucrose, 3 mM imidazole, pH 7.4) supplemented with protease/phosphatase inhibitors cocktail (see above). Next, cells were homogenized by 15–20 passages through a 22 G needle at 4 °C. Complete homogenization was confirmed under the phase microscope. The homogenate was centrifuged for 15 min at 1000*g* at 4 °C. The post-nuclear supernatant was collected and quantified by Bradford and 3 mg of PNS were brought to a final 40.2% sucrose (w/v) concentration by adding 2.5 M sucrose and loaded at the bottom of a 13.2-ml tube (Beckman UltraClear). Then 3 ml of 35% sucrose, 3 ml of 25% sucrose and 2.5 ml of homogenization buffer were overlaid stepwise on top. The gradient was centrifuged for 90 min at 150,000*g*, 4 °C in a Beckman SW 41 Ti rotor. After centrifugation, 1.5-ml fractions were collected from top to bottom and protein was precipitated using trichloroacetic acid/acetone to determine the TBC1D15 and Rab7 distribution by western blotting.

### Immunofluorescence

Cells grown on coverslips were fixed with 4% paraformaldehyde (PFA, Electron Microscopy Sciences) for 20 min at room temperature (RT), washed with PBS, permeabilized with 0.1% saponin for 10 min and blocked with 1% bovine serum albumin (BSA) for 5 min. Coverslips were incubated with primary antibody diluted in 0.02% saponin, 0.1% BSA in PBS for 1 h at RT, washed intensively and then incubated with the adequate secondary antibody labeled with Alexa Fluor-555 (Invitrogen) for 45 min at RT. After staining, coverslips were mounted in Mowiol (Calbiochem, Merck). Samples were visualized using a Leica TCS SP5 laser scanning confocal microscope equipped with a DMI6000 inverted microscope, blue diode (405 nm), Argon (458/476/488/496/514 nm), diode pumped solid state (561 nm), HeNe (594/633 nm) lasers and APO 63x oil immersion objective lens or a Leica DMI6000B epifluorescence inverted microscope equipped with an HCX PLA APO 63× oil immersion objective lens.

### LDL-cholesterol transport studies

To analyze the cellular fate of LDL-cholesterol, cells were plated on coverslips and grown in F-12 (HAM) supplemented with 5% LPDS for 48 h. Cells were then loaded with 50 µg/µl of LDL ± 10 µg/ml ACAT inhibitor (Sandow 58-035) for 24 h, fixed with 4% PFA for 1 h. Free cholesterol was stained with 0.05 mg/ml of filipin (Sigma Aldrich) and neutral lipids were stained with 1 µg/ml of BODIPY 493/503 (Molecular Probes) for 20 min at RT. Coverslips were mounted in Mowiol (Calbiochem, Merck). Alternatively, cellular cholesterol was stained with recombinant GST–PFO as follows: cells were fixed with 4% PFA for 15 min, permeabilized with 0.1% Triton X-100 (Sigma Aldrich) for 5 min and blocked with 3% fat free BSA (Sigma Aldrich) PBS for 30 min at RT. Cells were incubated with 10 µg/ml of purified recombinant GST–PFO in blocking buffer for 1 h at RT. Immunostaining with anti-GST (Abcam) and fluorescently labeled antibody was performed as above.

### Live-cell LDL-BODIPY-cholesteryl linoleate transport assay

NPC1-deficient CHO M12 cells seeded onto glass-bottom dishes (Nunc LabTek 4-well chambered coverglass) were transfected with non-targeting control and AnxA6 siRNAs in DMEM/F-12 supplemented with 5% LPDS. The transfections were carried with Lipofectamine RNAiMax (Thermo Scientific). 6 h later, 50 μg/ml Alexa Fluor 568-dextran (10,000 MW; Thermo Scientific) was added to the cells to label late endosomal organelles. 22 h after transfection, the cells were pulse-labeled for 2 h with 50 μg/ml BODIPY-cholesteryl linoleate-labeled LDL in serum-free medium, washed and chased in serum-free CO_2_-independent medium (Gibco) for the indicated times. Synthesis of BODIPY-cholesteryl linoleate was carried by Dr. Young Ah Kim (Queens College, New York) and labeling of human LDL was performed as previously described [55].

The chase was followed by confocal live-cell imaging on a Leica TCS SP8 attached to a motorized DMI 6000 inverted microscope with 63× HC PL APO CS2 water objective (1.20 NA). Experiments were performed at 37 °C in a fully enclosed temperature-controlled environmental chamber. Data were acquired with Leica LAS X (Leica Microsystems) and the efflux of late endosome BODIPY-cholesterol was quantified from background subtracted images with ImageJ by analyzing mean intensity of BODIPY-cholesterol fluorescence per cell.

For late endosome mobility analysis, time-lapse series were obtained with image acquisition frame rate of 370 ms. From the resulting live-cell videos, late endosome mobility was assessed by measuring the Pearson colocalization between subsequent frames/cell, and decreased degree of colocalization was considered indicative of increased late endosome mobility. Ten initial frames from time-lapse acquisitions were included in the analysis for each cell. All the data are expressed as mean ± SEM.

### Image analysis

Image analysis was performed with NIH ImageJ software [53]. When comparing different treatments, images were captured and systematically screened using identical microscope settings.

For number, size, fluorescence intensity and cellular distribution of late endosome and lipid droplets, a semi-automated ImageJ macro was designed and used. Specifically, fluorescence microscopy images were locally thresholded and vesicles were selected through the ImageJ particle analysis function. Number, size and fluorescence intensity from raw images were then calculated. Cellular distribution was analyzed using the 3D ImageJ Suite [61].

### Electron microscopy

For conventional electron microscopy, pellets from fractions enriched with late endosomes from discontinuous sucrose gradients or cells in culture were washed in PBS and fixed overnight (gradient fractions) or for 1 h (cells) in 2.5% glutaraldehyde in 0.1 M phosphate buffer (PB) at RT. Next, samples were slowly and gently scraped and pelleted in 1.5 ml tubes. Pellets were washed in PB and incubated with 1% OsO_4_ for 90 min at 4 °C. Then samples were dehydrated, embedded in Spurr and sectioned using Leica ultramicrotome (Leica Microsystems). Ultrathin sections (50–70 nm) were stained with 2% uranyl acetate for 10 min, a lead-staining solution for 5 min and observed using a transmission electron microscope, JEOL JEM-1010 fitted with a Gatan Orius SC1000 (model 832) digital camera.

Perimeter and areas of contact between late endosome/lysosomes (LE/Lys) and ER–LE/Lys were identified by morphology was measured with ImageJ [53]. At least 20–50 cells were analyzed per experiment and data were analyzed from duplicate or triplicate separate experiments. At least two grids were used for each condition. The minimum number of cells scored for each condition was 25 and the average number of sections (fields) 40.

### Statistical analysis

Unless mentioned in the figure legend, group data are presented as mean ± SD. Comparison between 2 groups were analyzed by Student’s *t* test; comparison between more than 2 groups were analyzed by one-way ANOVA with a Bonferroni post hoc test, and comparison between groups and condition were analyzed by Bonferroni post-tested two-way ANOVA for condition and group differences using GraphPad Prism software. Differences were considered statistically significant at *p *< 0.05. *p < 0.05, **p < 0.01, ***p < 0.001.

## Results

### Interaction of AnxA6 with the Rab7-GAP TBC1D15

We previously demonstrated that AnxA6 overexpression led to late endosome-cholesterol accumulation, a phenotype reminiscent of the NPC1 mutant phenotype [46, 62]. This was accompanied by an increased recruitment of AnxA6 to cholesterol-laden late endosomes upon pharmacological NPC1 inhibition, using U18666A, or loading with LDL [43–45]. In order to unravel the underlying mechanism, we reasoned that cells lacking NPC1-dependent cholesterol export pathways would be the most promising model to address how AnxA6 could affect late endosome-cholesterol levels.

**Fig. 1:**
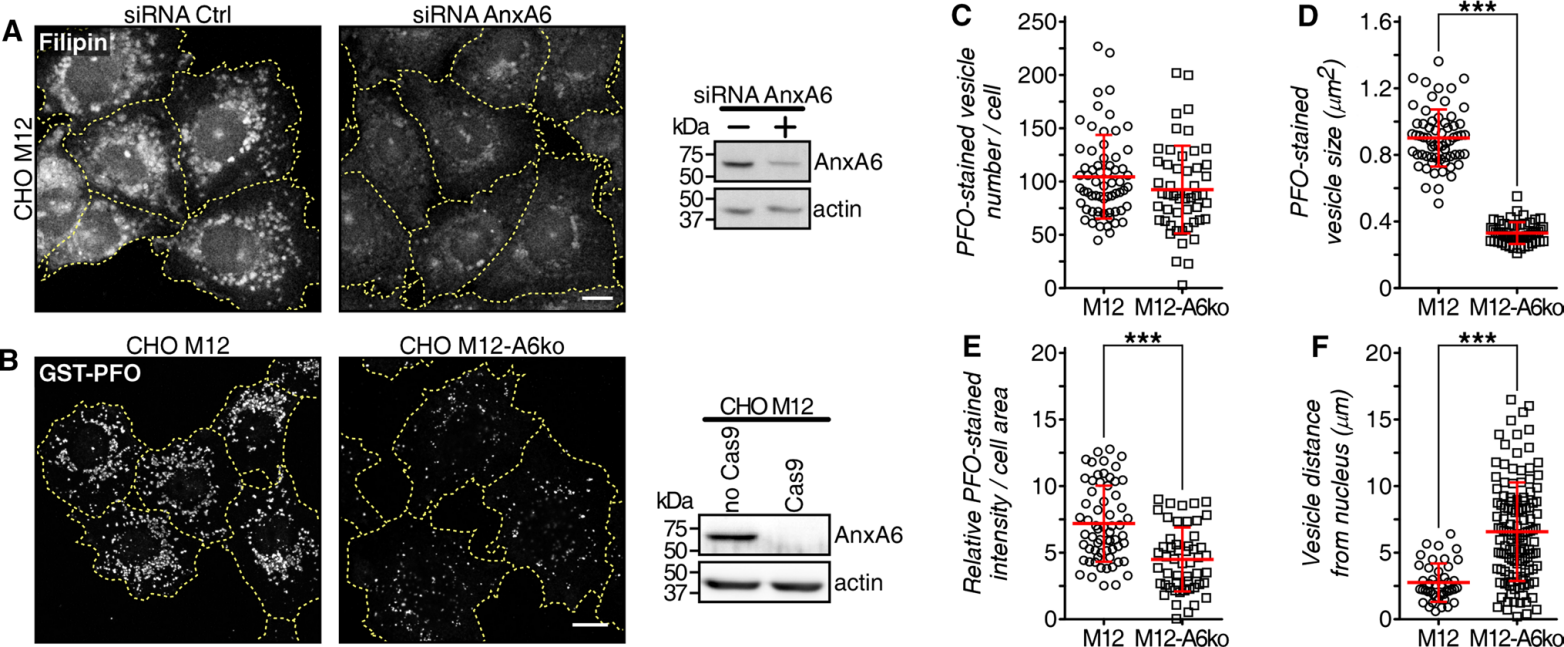
AnxA6 depletion rescues late endosomal cholesterol accumulation in NPC1 mutant cells. **a** CHO M12 cells expressing control siRNA (siRNA Ctrl) or siRNA targeting AnxA6 (siRNA AnxA6) were fixed and stained with filipin. For better comparison of filipin staining, the outline and shape of transfected cells is indicated. AnxA6 protein levels in lysates from CHO M12 cells ± siRNA AnxA6 is shown. Scale bar, 10 μm. **b** Stable AnxA6 gene deletion in CHO M12 cells (CHO M12-A6ko) using CRISPR/Cas9 genome editing technology. Cholesterol was visualized using GST–perfringolysin O (PFO) staining. For better comparison of GST–PFO staining, the outline and shape of cells is indicated. Representative cells and AnxA6 protein levels in lysates from CHO M12 and CHO M12-A6ko cells are shown. Scale bar, 10 μm. **c–f** Dot-plot of PFO-stained vesicle number (**c**), size (**d**), PFO relative staining intensity (**e**) and vesicle distance to the nucleus (**f**) of a representative experiment from CHO M12 and CHO M12-A6ko cells (*n* > 60 cells, 3 experiments). For quantification details see “Materials and methods”. ***p < 0.001 by two-tailed Student’s *t* test (**c–f**). All data are presented as mean ± SD in red

Most strikingly, in the NPC1 mutant cell line CHO M12, depleting AnxA6 using a small interfering RNA (siRNA) or CRISPR/Cas9 technology (CHO M12-A6ko) led to a significant reduction of late endosome-cholesterol accumulation in the perinuclear region, determined using both filipin and PFO labeling to visualize unesterified (free) cholesterol [63] (Fig. 1a, b; quantified in c–e). In addition, loss of late endosome-cholesterol accumulation in AnxA6-depleted CHO M12 cells was associated with increased late endosome positioning towards the cell periphery (Fig. 1f), a feature common to late endosomes with normal cholesterol content.

We performed a yeast two-hybrid screen (Hybrigenics Services, France) using the N-terminal region of human AnxA6 (aa1–273) as bait against a cDNA library from human liver to identify AnxA6 interaction partners that could explain the rescue of the NPC1 mutant phenotype upon AnxA6 depletion. These studies identified TBC1D15, a Rab7-GAP [64, 65–69], as a possible AnxA6-binding protein.

**Fig. 2:**
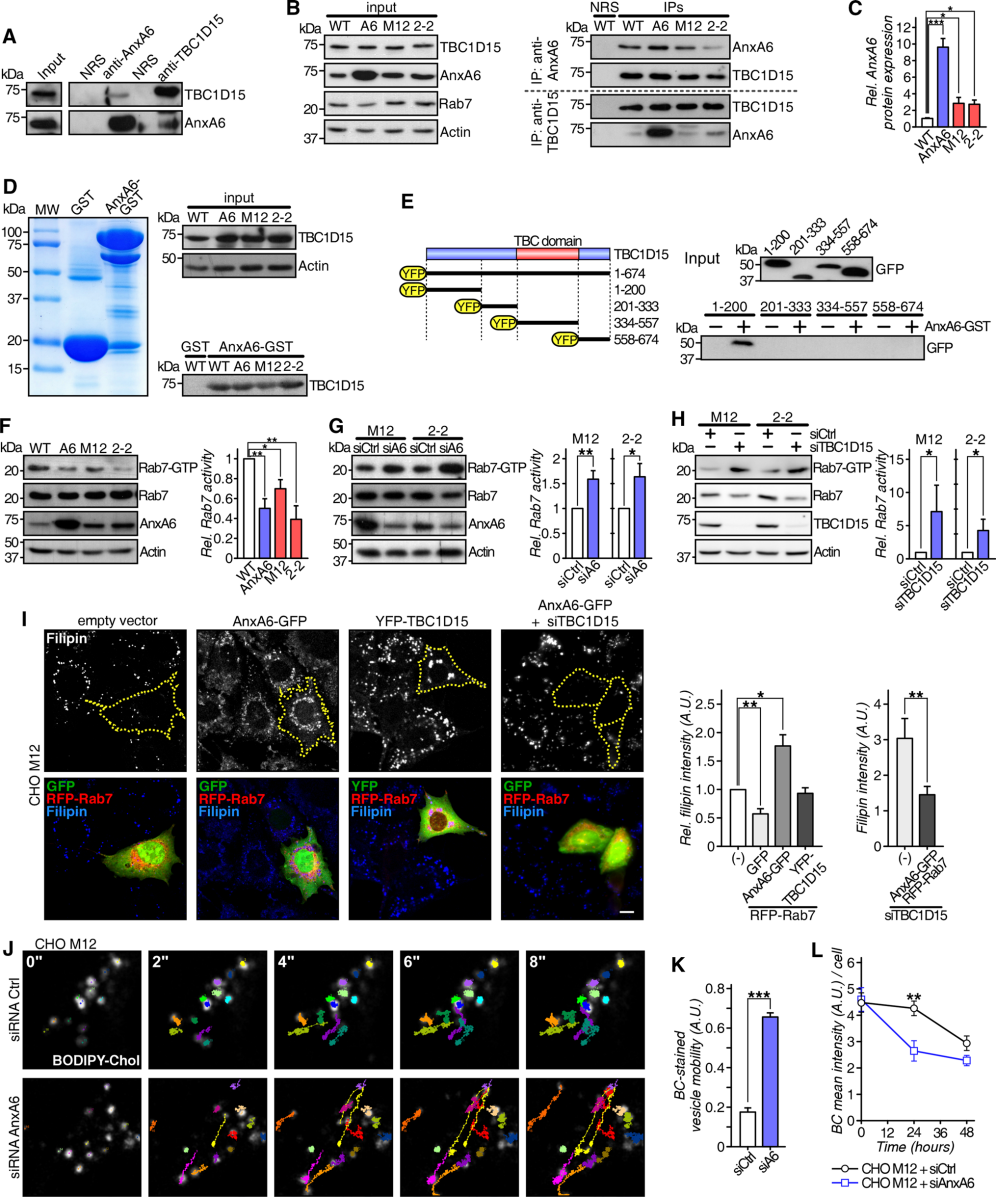
AnxA6 interacts with TBC1D15. **a** Mouse liver homogenates were immunoprecipitated with antibodies against AnxA6, TBC1D15 or control antibody (normal rabbit serum, NRS) and analyzed by western blotting for co-immunoprecipitation. Representative images of AnxA6 and TBC1D15 levels (5% of total input) and immunoprecipitations are shown (*n* = 2). **b** Cell lysates from CHO-WT, CHO-A6, CHO M12 and CHO 2-2 cells were immunoprecipitated with antibodies against AnxA6, TBC1D15 or control antibody (NRS) as indicated and analyzed by western blotting for co-immunoprecipitation. Representative images of TBC1D15, AnxA6, Rab7 and actin levels in the cell lysates (10% of total input, left panel) and immunoprecipitations are shown (*n* = 3). **c** Quantification of AnxA6 levels in CHO-WT, CHO-A6, CHO M12 and CHO 2-2 cell lysates (*n* = 3). **d** Coomassie blue staining of purified glutathione S-transferase (GST) and GST–AnxA6 used in the pull-down assays shown in D–E. Cell lysates (2% of total input) from CHO-WT, CHO-A6, CHO M12 and CHO 2-2 cells were incubated with GST or GST–AnxA6 fusion protein in pull-down assays and analyzed by western blotting with anti-TBC1D15 as indicated (*n* = 3). **e** Scheme of YFP-tagged TBC1D15 wild type (1–674aa) and deletion mutants (1–200, 201–333, 334–557, 558–674) used to map the interaction of TBC1D15 with AnxA6. The TBC domain in TBC1D15 (334–557) is indicated. Cell lysates from COS-1 cells ectopically expressing YFP–TBC1D15 wild type and mutants were incubated ± GST–AnxA6 fusion protein in pull-down assays and analyzed by western blotting for interaction with anti-GFP as indicated. Expression levels of YFP-tagged TBC1D15 deletion mutants (5% of total input) in COS-1 cell lysates are shown (*n* = 2). **f** Cell lysates from CHO-WT, CHO-A6, CHO M12 and CHO 2-2 cells were subjected to Rab interacting lysosomal protein (RILP)-C33–GST pull-down assays to determine active Rab7 (Rab7-GTP) levels. **g** Rab7-GTP levels determined as above with lysates from CHO M12 cells expressing non-targeting control siRNA (siCtrl) or siRNA targeting AnxA6 (siA6). Total levels of Rab7, AnxA6, and actin in cell lysates and the quantification of relative Rab7 activity are shown (5% of total input, *n* = 3). **h** Representative western blot showing Rab7-GTP levels determined as above with lysates from CHO M12 and CHO 2-2 cells expressing non-targeting control siRNA (siCtrl) or siRNA targeting TBC1D15 as indicated. Total levels of Rab7, TBC1D15 and actin in cell lysates (5% of total input) and the quantification of relative Rab7 activity are shown (*n* = 3). **i** CHO M12 cells were co-transfected with RFP–Rab7 (red) and empty vector (GFP), AnxA6–GFP, YFP–TBC1D15 or AnxA6–GFP together with siRNA targeting TBC1D15 (siTBC1D15) (green) as indicated. Cells were fixed and stained with filipin (blue). For better comparison of filipin staining, the outline and shape of transfected cells is indicated. Merged images are shown. Scale bar, 10 μm. The mean relative filipin intensity of at least 20 transfected cells from 3 independent experiments was quantified (*n* = 3). **j** Confocal images of CHO M12 cells expressing control siRNA (siRNA Ctrl) or siRNA targeting AnxA6 (siRNA AnxA6). Cells were grown in lipoprotein-protein deficient serum (LPDS), and then pulse-labeled with LDL-BODIPY-cholesteryl linoleate (see “Materials and methods” for details). Vesicles labeled with LDL-derived BODIPY-cholesterol (BC) were imaged in live cells over time. Representative images tracing pseudocoloured individual BC-stained vesicles for CHO M12 ± AnxA6 are shown (0–8 s) after 24 h chase. For representative frames from live-cell videos see Movies S1 and S2. **k** Quantitation of BC-stained vesicle mobility in CHO M12 ± AnxA6 (siCtr, siA6) after pulse-labeling with LDL-BODIPY-cholesteryl linoleate, followed by 24 h chase. Bars: 1 − [Pearson’s colocalization coefficient between subsequent frames/cell] ± SEM (*n* = 24–26 cells, two experiments). **l** Analysis of late endosomal (LE) BODIPY-cholesterol removal. CHO M12 cells were transfected with control siRNA (siCtrl) or siRNA targeting AnxA6 (siAnxA6). 22 h after transfection, cells were pulse-labeled for 2 h with 50 μg/ml LDL-BODIPY-cholesteryl linoleate. Cells were washed and chased in medium with 5% lipoprotein-deficient serum for 0–48 h. The efflux of LDL-derived BODIPY-cholesterol was quantified by analyzing mean fluorescence intensity per cell (*n* = 31–33 cells, two experiments). *p < 0.05; **p < 0.01; ***p < 0.001 by one-way ANOVA with Bonferroni post hoc test (**c**, **f**, **i**), two-tailed Student’s *t* test (**g–i**, **k**) or two-way ANOVA with Bonferroni post hoc test (**l**). All data are shown as mean ± SEM

Previous reports suggested that ectopic expression of Rab7 could restore cholesterol re-esterification and neutral lipid deposition in NPC1 mutants [36]. Therefore, the potential interaction of AnxA6 with a Rab7-GAP was pursued further. Reciprocal co-immunoprecipitations with antibodies against AnxA6 and TBC1D15 confirmed the interaction between AnxA6 and TBC1D15 in mouse liver homogenates (Fig. 2a), cell lysates from CHO-WT, AnxA6 overexpressing CHO (CHO-A6) [43], and NPC1 mutant cell lines CHO M12 and CHO 2-2 (Fig. 2b). Notably, AnxA6 protein levels were elevated in NPC1 mutant cells (CHO M12, CHO 2-2), compared with CHO-WT cells (Fig. 2c) (see “Discussion”). Pull-down assays with AnxA6–GST fusion protein further established the ability of AnxA6 to directly interact with TBC1D15 (Fig. 2d) and use of truncated TBC1D15 mutants mapped the interaction of AnxA6 to the N-terminal (1–200aa) region of TBC1D15 (Fig. 2e).

We next addressed a possible involvement of AnxA6/TBC1D15 interaction in Rab7-mediated late endosome-cholesterol egress. AnxA6 depletion in CHO M12 cells significantly reduced the association and colocalization of YFP–TBC1D15 with late endosome and lysosome structures expressing the constitutively active Rab7 mutant GFP–Rab7-Q67L (Fig. S1a). In CHO M12 cells, quantification showed that 68 ± 18% of YFP-labeled ring structures colocalized with GFP-positive vesicles (see also line profile in Fig. S1a). In contrast, CHO M12-A6ko cells contained significantly fewer GFP-positive vesicles that colocalized with YFP-labeled ring formations (36 ± 23%).

To provide additional evidence for AnxA6-regulated association of TBC1D15 with late endosomes, subcellular fractionation was performed to compare the cellular distribution of TBC1D15 in CHO-WT and CHO-A6 cells (Fig. S1b). Significant enrichment of TBC1D15 in the late endosome fraction (F2) of CHO-A6 cells was observed (Fig. S1b). Electron microscopy of the late endosome fraction from CHO cells (Fig. S1c) detected vesicular structures, sometimes with internal membrane fragments and/or multilamellar prototypical endolysosomes. Importantly, mitochondria, peroxisomes or microsomes (ER) were completely absent from this fraction. These data suggested that AnxA6 targets TBC1D15 to Rab7-positive endosomes, and correlated with reduced filipin staining in AnxA6-depleted CHO M12 cells (Fig. 1). Thus, high levels of AnxA6 appear to induce complex formation with TBC1D15 and its recruitment to Rab7-GTP-positive late endosomes and lysosomes, and removal of the AnxA6/TBC1D15 complex to restore late endosome-cholesterol export in NPC1 mutants.

### AnxA6 interferes with Rab7 activity to impair late endosome-cholesterol egress

To determine whether increased AnxA6 levels could promote Rab7-GAP activity, we examined Rab7-GTP levels using RILP-C33–GST pull-down assays (Fig. 2f–h) in cell lysates containing moderate (CHO-WT) or elevated (CHO-A6, M12, 2-2) AnxA6 protein levels (see Fig. 2c). In addition, we also compared Rab7-GTP amounts in A431wt cells that lack endogenous AnxA6, with those from a well-characterized A431 line stably expressing AnxA6 (A431-A6) (Fig. S2a) [50, 70], as well as in MEFs from wild type (MEF-WT) and AnxA6-KO (MEF-A6ko) mice (Fig. S2b) [40, 51]. Both A431-A6 and MEF-WT express AnxA6 levels commonly found in other cell lines and tissues [50, 70]. Indeed, cells with elevated AnxA6 protein expression displayed a substantial and significant reduction in Rab7-GTP levels (Fig. 2f). On the other hand, AnxA6 knockdown in the NPC1 mutant CHO M12 or CHO 2-2 cells was associated with effectively increased Rab7-GTP levels (Fig. 2g). Furthermore, TBC1D15 depletion strongly increased Rab7-GTP amounts in NPC1 mutant M12 and 2-2 cells (Fig. 2h). Thus, elevated AnxA6 levels create an environment that favors TBC1D15-mediated Rab7 inactivation. These studies reveal for the first time that AnxA6 can regulate Rab7 GTPase via direct binding and recruitment of a member of the TBC/Rab7GAP-family, TBC1D15 to late endosomes, thereby inhibiting Rab7 activity.

To further confirm that AnxA6- and/or TBC1D15-induced downregulation of Rab7-GTP levels would interfere with the ability of overexpressed Rab7 to rescue the NPC1 mutant phenotype [36], RFP-Rab7 was ectopically co-expressed with GFP, AnxA6–GFP or YFP–TBC1D15 in NPC1 mutant CHO M12 cells. To visualize late endosome-cholesterol accumulation, cells were fixed and stained with filipin (Fig. 2i). In agreement with previous data [36], transient overexpression of Rab7 drastically reduced late endosome-cholesterol accumulation in CHO M12 cells, yet co-expression of Rab7 with AnxA6 or TBC1D15 not only blocked Rab7-mediated rescue of late endosome-cholesterol export (YFP–TBC1D15), but also increased late endosome-cholesterol accumulation (AnxA6–GFP). Strikingly, TBC1D15 depletion in CHO M12 cells restored late endosome-cholesterol export even upon ectopic co-expression of AnxA6–GFP (Fig. 2i). These findings show unequivocally that AnxA6, acting through TBC1D15, reduces Rab7-GTP and blocks late endosome-cholesterol egress in NPC1 mutant cells. In support of this, expression of the constitutively active Rab7 mutant Rab7-Q67L was sufficient to release accumulated late endosome-cholesterol in CHO M12 cells (Fig. S2c). Moreover, ectopic expression of the YFP–TBC1D15_(1–200)_ deletion mutant, which still interacts with AnxA6 (see Fig. 2e), yet lacks the GAP domain to inactivate Rab7 (and therefore acts as a dominant-negative mutant), also showed a significant reduction of late endosome-cholesterol in CHO M12 cells (Fig. S2d). In contrast, ectopic expression of the dominant-negative GFP–Rab7-T22N mutant inhibited late endosome-cholesterol egress in AnxA6-depleted CHO M12 cells, demonstrating the requirement for active Rab7 protein for the rescue of the NPC1 mutant phenotype (Fig. S2d). Collectively, these observations clearly indicate that AnxA6 modulates late endosome-cholesterol levels by regulation of Rab7 activity.

Importantly, in line with elevated Rab7 activity (Fig. 2f) and increased late endosome positioning towards the cell periphery (Fig. 1f) in AnxA6-depleted M12 cells, we observed the re-establishment of late endosome motility as judged by live-cell microscopy of M12 cells labeled with LDL-derived BODIPY-cholesterol upon AnxA6 depletion (Fig. 2j; quantified in k and Movies S1 and S2). One outcome of this stimulated late endosome-trafficking could be the redistribution of late endosome-cholesterol to other destinations. Indeed, analysis of BODIPY-cholesterol efflux from late endosomes in the presence of extracellular cholesterol acceptors revealed a faster removal of BODIPY-cholesterol from AnxA6-depleted CHO M12 (siRNA-AnxA6) cells compared with control cells (Fig. 2l). Previous studies implicated ectopic and non-physiological elevation of Rab7 levels as a prerequisite to restore neutral lipid storage and cholesterol esterification in NPC1 mutants [36]. However, our studies strongly suggest that elevation of endogenous Rab7-GTP levels, through the depletion of AnxA6 (or TBC1D15), is sufficient to re-establish the ability of NPC1 mutant cells to export late endosome-cholesterol to the cell surface (Fig. 2l), or store as neutral lipid in lipid droplets (see below).

### AnxA6 depletion restores cholesterol trafficking in NPC1 mutant cells

In wild type cells, endocytosed, esterified LDL-cholesterol is hydrolyzed into free cholesterol in late endosomes and lysosomes, to be delivered to other cellular sites, including the ER for re-esterification and subsequent storage as cholesteryl esters in lipid droplets [3, 5]. In NPC1 mutant cells, late endosome-cholesterol accumulation is accompanied by reduced cholesterol re-esterification and neutral lipid deposition [36]. To address the trafficking of late endosome-cholesterol in AnxA6-depleted CHO M12 cells, we set up experimental conditions to monitor the trafficking of LDL-derived cholesterol out of late endosomes and lysosomes. CHO-WT and NPC1 mutant cells were grown in LPDS-containing media for 48 h before loading with LDL for additional 24 h (Fig. S3a) [38]. After 48 h, a significant reduction of filipin and neutral lipid stain (BODIPY 493/503-positive structures, green) in both CHO-WT and NPC1 mutant cell lines (CHO M12) was observed, pointing to strongly reduced late endosome-cholesterol and neutral lipid levels in lipid droplets. As expected, subsequent LDL loading caused late endosome-cholesterol accumulation only in CHO M12 cells, while CHO-WT cells showed cholesterol redistribution and robust BODIPY staining (Fig. S3b).

**Fig. 3:**
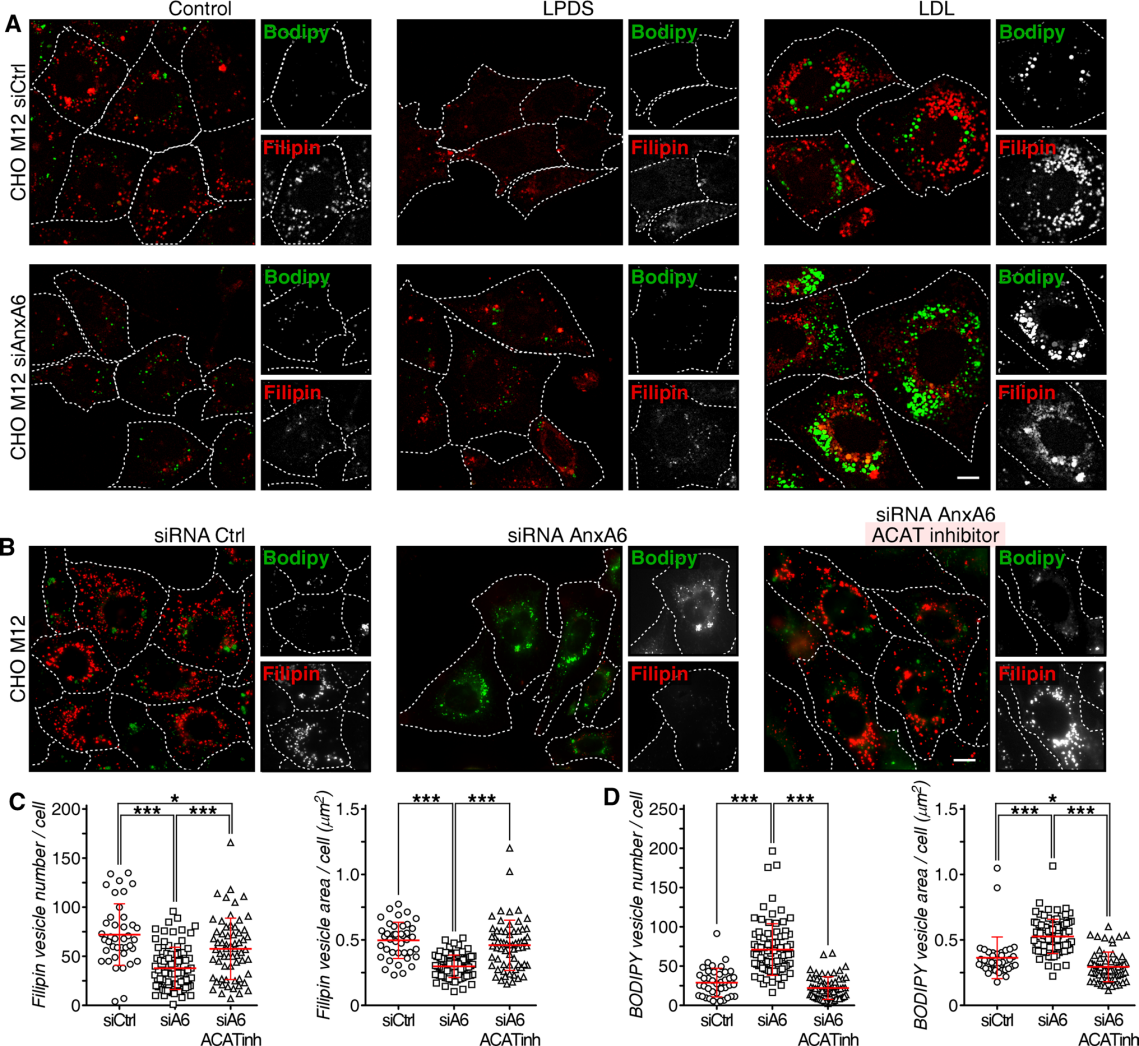
AnxA6 knockdown elicits late endosome-cholesterol release and neutral lipid accumulation in NPC1 mutant cells. **a** CHO M12 cells expressing control siRNA (siCtrl) or siRNA targeting AnxA6 (siAnxA6) were grown in 10% fetal calf serum (0 h, control), then starved in 5% lipoprotein-deficient serum (LPDS) for 48 h before loading with 50 µg/ml LDL (± 10 μg/ml ACAT inhibitor Sandoz 58–035 in **b**) for 24 h (see scheme in Fig. S3a, and “Materials and methods” for details). At each time point (0, 48 and 72 h), cells were fixed, stained with filipin (cholesterol, red) and BODIPY 493/503 (neutral lipids, green). Representative fields of cells at *t* = 0 (control), *t* = 48 (LPDS) and *t* = 72 h (LDL) (merged and split channels) are shown. Enlarged regions of interest are shown. For better comparison of filipin and BODIPY staining, the outline and shape of cells is indicated. Scale bar, 10 μm. A representative western blot showing siRNA AnxA6 depletion in CHO M12 cells is provided. Actin served as loading control. **c–d** Dot-plot of number and area of filipin-stained (LE) and BODIPY-stained (LD) vesicles per cell of a representative experiment (*n* > 60, 3 experiments). For quantification details see “Materials and methods”.*p < 0.05; ***p < 0.001 by one-way ANOVA with Bonferroni post hoc test (**d**, **e**). All data are presented as mean ± SD in red

Given that AnxA6 depletion reduced perinuclear late endosome-cholesterol accumulation in CHO M12 cells (Fig. 1), we next investigated if the loss of AnxA6 could also rescue the delivery of late endosome-cholesterol for neutral lipid storage in lipid droplets in these cells. Indeed, compared with a control siRNA (siCtrl), AnxA6 depletion not only reduced late endosome-cholesterol accumulation, as judged by reduced filipin staining (quantified in Fig. S4a), but significantly increased neutral lipid stain (BODIPY-positive structures, green) in CHO M12 cells (Fig. 3a; quantified in c and d). A slightly increased number of lipid droplets was observed under normal growth conditions in AnxA6-depleted CHO M12 cells (Fig. S4a). Colocalization with anti-adipophilin, a well-established lipid droplet marker [71], confirmed the identity of these structures in AnxA6-depleted CHO M12 cells (Fig. S4b). Consistent with our previous results (Fig. 1), decreased filipin staining was evident in CHO M12-A6ko cells (quantified in Fig. S4a). Furthermore, and in strong support of these findings, conventional electron microscopy showed abundant lipid droplets after LDL loading not only in CHO-WT, but also in CHO M12-A6ko cells; in contrast, as expected [36], very few lipid droplets were observed in LDL-loaded CHO M12 cells (Fig. S4c; see quantification).

These results indicate that AnxA6 depletion overcomes defective transport of LDL-cholesterol to the ER in NPC1 mutants. Increased cholesterol levels in the ER suppress processing of the major transcriptional regulator of cholesterol homeostasis, sterol regulatory element binding protein 2 (SREBP2), enabling mature SREBP (mSREBP2) to enhance the transcription of its target genes [72]. In line with published data [73], LDL-cholesterol failed to suppress SREBP2 maturation in NPC1 mutants. However, AnxA6 depletion in CHO M12 was associated with decreased amounts of mSREBP2 upon LDL loading (Fig. S5). To further verify that late endosome-cholesterol delivery to the ER, followed by ACAT-mediated cholesterol esterification, would drive transfer of neutral lipids into newly formed lipid droplets in AnxA6-depleted CHO M12 cells, a pharmacological ACAT inhibitor (Sandoz 58-035) was employed [74]. ACAT inhibition completely abrogated neutral lipid (cholesteryl ester) accumulation and lipid droplet formation in AnxA6-depleted M12 cells, while a concomitant increase of filipin staining was observed (Fig. 3b; quantified in c and d).

Finally, employing the same experimental conditions described above (Fig. 3a), we addressed if TBC1D15 depletion would also impact the transfer of neutral lipids into lipid droplets in NPC1 mutant CHO M12 cells. Indeed, consistent with TBC1D15 silencing leading to elevated Rab7-GTP levels (Fig. 2h) and restoration of late endosome-cholesterol efflux (Fig. 2i), depletion of TBC1D15 in CHO M12 cells was accompanied with increased BODIPY-positive lipid droplet numbers (Fig. S2e, f; quantified in g and h). Thus, both TBC1D15 and AnxA6 depletion enabled neutral lipid storage in LDL-loaded CHO M12, confirming the hypothesis that both proteins contribute to a role for Rab7 in cholesterol homeostasis. Taken together, these data indicate that AnxA6 as well as TBC1D15 deficiency in NPC1 mutant cells promotes transport of free cholesterol from late endosomes to lipid droplets in an ACAT-dependent manner.

### StARD3 is required to rescue late endosome-cholesterol export in NPC1 mutant cells lacking AnxA6

It was previously reported that enlarged and cholesterol-laden late endosomes have impaired vesicular trafficking [35]. In addition, active Rab7 is required to promote MCS formation between late endosomes and lysosomes and the ER [12], providing protein–protein interactions within MCS for the bidirectional transfer of cholesterol and other lipids between late endosomes and ER [75, 76]. Given elevated Rab7-GTP levels and increased late endosome motility in AnxA6-depleted NPC1 mutant cells (Fig. 2), we reasoned that increased MCS formation could aid cholesterol transfer in these cells. However, CHO M12 lack NPC1 and do not express ORP1L (Fig. S6), excluding the ORP1L/VAP-A-dependent cholesterol transfer route [4, 6, 15, 16, 77–79].

**Fig. 4:**
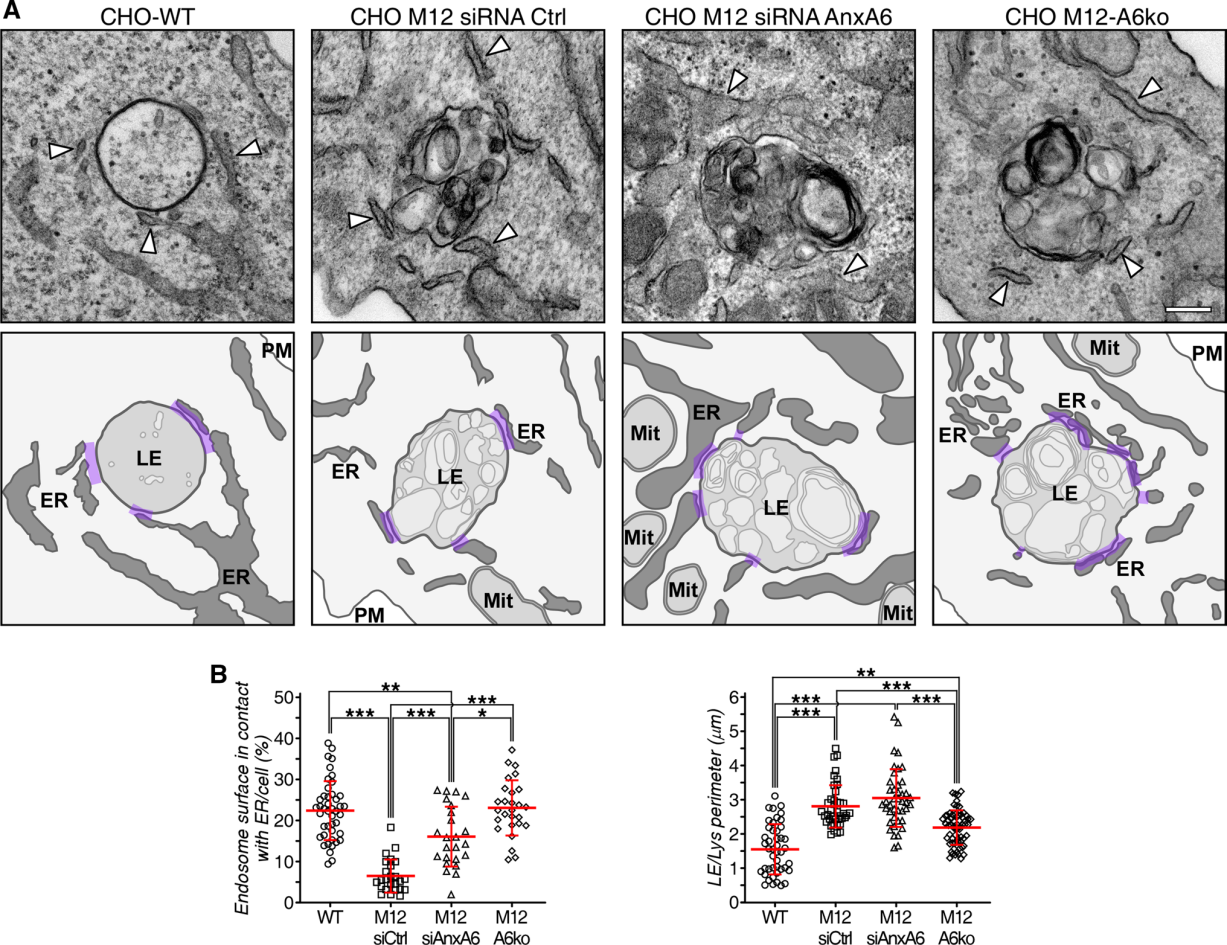
Increased membrane contact sites in CHO M12 cells after AnxA6 depletion. **a** Representative transmission electron microscopy (TEM) images of late endosomes/lysosomes (LE/Lys) from CHO-WT, CHO M12 expressing control siRNA (siRNA Ctrl), siRNA targeting AnxA6 (siRNA AnxA6) and CHO M12-A6ko cells. Arrowheads indicate endoplasmic reticulum (ER). A schematic representation of these images with highlighted membrane contacts (purple) between ER and LE/Lys structures is shown. **b** Quantitative stereology of ER–LE/Lys contacts in TEM sections is given: perimeter of LE/Lys contacts (in microns) and percentage of endosome surface in contact with the ER per cell (*n* > 30). *LE* late endosomes, *ER* endoplasmic reticulum, *Mit* mitochondria, *PM* plasma membrane. Scale bar, 200 nm. *p < 0.05; ***p < 0.001 by one-way ANOVA with Bonferroni post hoc test. All data are presented as mean ± SD in red

We examined the impact of AnxA6 depletion on MCS between late endosomes/lysosomes and the ER in CHO M12 cells. Since the size of MCS is in the nanometer range (~ 5–30 nm), electron microscopy is currently the best approach to identify and quantify these membrane domains. Therefore, CHO-WT, CHO M12 cells with and without AnxA6 were prepared for conventional electron microscopy and ultrathin sections were analyzed and images quantified using ImageJ. For quantifications, we selected vesicle sections harboring prototypical late endosomes and lysosomes with electron-dense membranes of variable size containing bits of electron-dense material [80], similar to those structures observed by others after treatment with U18666A. Figure 4a shows representative late endosome images from CHO-WT, CHO M12 and AnxA6-depleted CHO M12 cells, where the surface contacts between late endosomes and the ER were quantified by stereology. These data sets show reduced MCS formation or stability of NPC1-mutant CHO cells and clearly demonstrate that AnxA6 depletion increased MCS formation in CHO M12 cells. In fact, this is the first quantitative study showing diminution of MCS in NPC1 mutant cells (Fig. 4b).

**Fig. 5:**
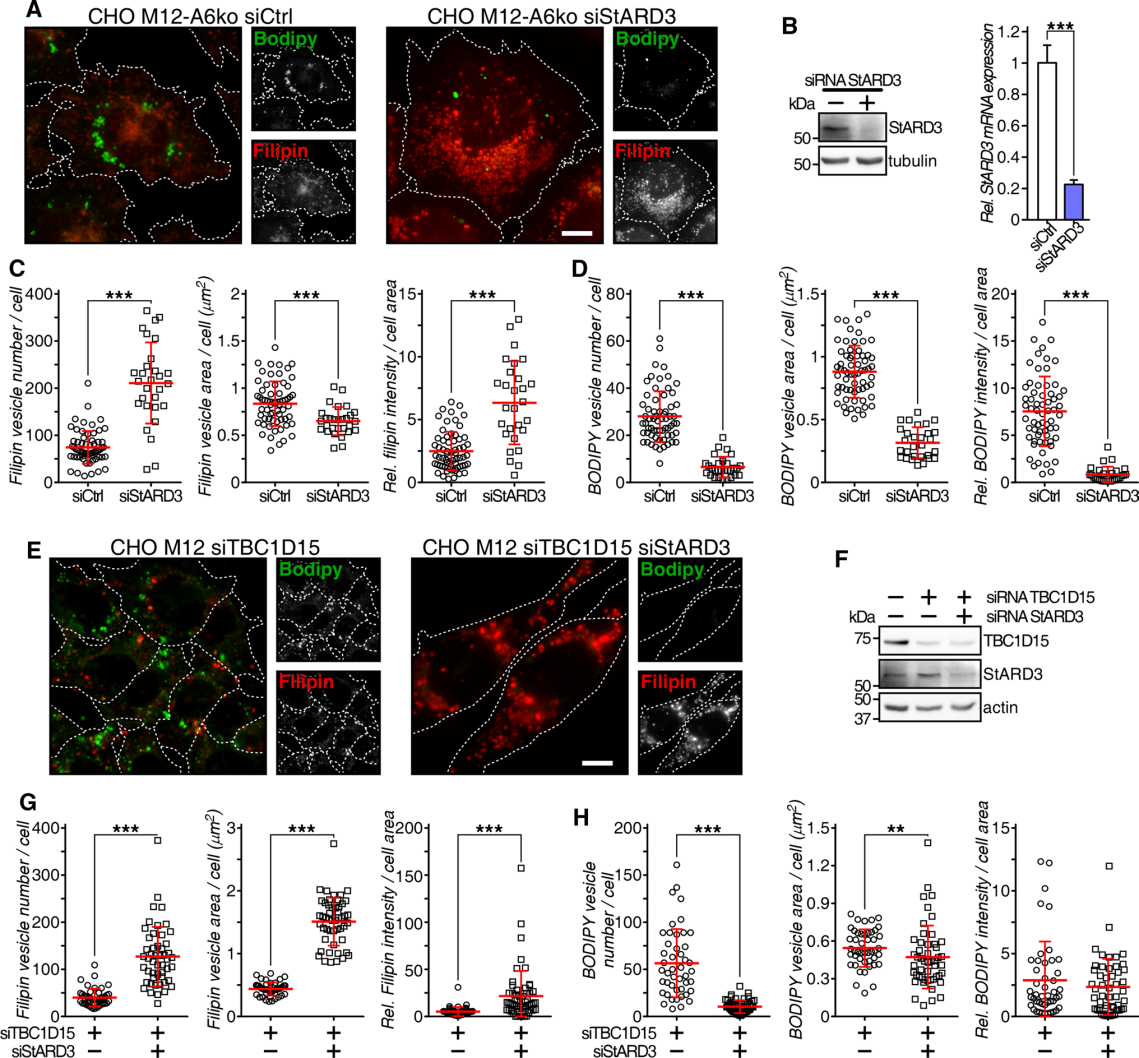
StARD3 contributes to late endosome-cholesterol egress and neutral lipid accumulation in AnxA6-deficient NPC1 mutant cells. **a** CHO M12-A6ko cells expressing control siRNA (siCtrl) or siRNA targeting StARD3 (siStARD3) were starved in 5% LPDS for 48 h before loading with 50 µg/ml LDL for 24 h. Cells were fixed and stained with filipin (cholesterol, red) and BODIPY 493/503 (neutral lipids, green). Enlarged regions of interest are shown. For better comparison of filipin and BODIPY staining, the outline and shape of cells is indicated. Scale bar, 10 μm. **b** Western blot analysis (StARD3, tubulin) and RNA quantification determined by qPCR (*n* = 3) of StARD3 knockdown in CHO M12-A6ko cells is shown. **c–d** Dot-plot of number, area and relative intensity of filipin-stained (LEs) and BODIPY-stained (LDs) vesicles per cell of a representative experiment (*n* > 60, 3 experiments). For quantification details see “Materials and methods”. **e** CHO M12 cells expressing control siRNA (siCtrl) or siRNA targeting TBC1D15 and StARD3 (siTBC1D15 siStARD3) were starved in 5% LPDS for 48 h and loaded with 50 µg/ml LDL for 24 h. Then cells were fixed, stained with filipin (cholesterol, red) and BODIPY 493/503 (neutral lipids, green), and representative fields (merged and split channels) are shown. Enlarged regions of interest are shown. For better comparison of filipin and BODIPY staining, the outline and shape of cells is indicated. Scale bar, 10 μm. **f** Western blot analysis of TBC1D15, StARD3 and actin ± siRNA-mediated TBC1D15 and StARD3 knockdown from lysates of CHO M12 cells as indicated. **g–h** Dot-plot of number, area and relative intensity of filipin-stained (LE) and BODIPY-stained (LD) vesicles per cell of a representative experiment in CHO M12 cells ± siTBC1D15 and siStARD3 as indicated (*n* > 60, 3 experiments). For quantification details see “Materials and methods”.**p < 0.01; ***p < 0.001 by two-tailed Student’s *t* test (**b**–**d**, **g**, **h**). Data are shown as mean ± SEM (**b**) or as mean ± SD in red (**c**, **d**, **g**, **h**)

Finally, we investigated whether the StARD3/VAP-A protein complex that can facilitate cholesterol transfer between late endosomes and the ER [75, 76] could contribute to the rescue of late endosome-cholesterol export in CHO M12-A6ko cells. The ability of AnxA6 depletion to reduce late endosome-cholesterol accumulation in CHO M12 cells was lost upon StARD3 depletion and concomitantly, strongly interfered with neutral lipid accumulation in lipid droplets of CHO M12-A6ko cells (Fig. 5a, quantified in c and d; also, electron microscopy in Fig. S4c). This observation was accompanied by a wide distribution of LDL-containing and filipin-positive late endosomes (Fig. 5a), as shown previously for StARD3-deficient HeLa cells [23]. Consequently, AnxA6 depletion rescued late endosome-cholesterol accumulation in NPC1 mutant cells via StARD3-dependent cholesterol transport routes. Further supporting a role for StARD3 downstream of the AnxA6–TBC1D15–Rab7 axis, simultaneous depletion of TBC1D15 and StARD3 in CHO M12 cells was associated with late endosome-cholesterol accumulation in more scattered late endosomes (Fig. 5e). Hence, despite upregulated Rab7 activity due to TBC1D15 depletion, StARD3 depletion interfered with late endosome-cholesterol egress and consequently, these cells did not display lipid droplets (Fig. 5e; quantified in g and h). In strong support of these findings, and in line with published data [36], conventional electron microscopy showed abundant lipid droplets in LDL-loaded CHO-WT cells, but not CHO M12 cells. Lipid droplet formation upon LDL loading was restored in AnxA6-depleted CHO M12 cells, yet depletion of StARD3 in these cells resulted in a lack of lipid droplets, further validating the requirement of StARD3 to store LDL-derived neutral lipids as cholesteryl esters (Fig. S4c and quantification). Altogether, AnxA6 deficiency in CHO M12 cells correlated with a significant increase of surface contacts between late endosomes and the ER (MCS), possibly facilitating the transfer of cholesterol out of late endosomes.

Although it was suggested that non-functional NPC1 may impair MCS formation between ER and late endosomes [13], this has not been proven experimentally. Here we identified decreased MCS numbers in LDL-loaded NPC1 mutant CHO M12 cells compared with CHO-WT cells (Fig. S4d) (CHO-WT cells contain less AnxA6 than CHO M12; see Fig. 2c). However, those MCS numbers were significantly expanded by the depletion of AnxA6 in NPC1 mutant cells, creating an increased surface contact between late endosomes and the ER. More remarkably, in these settings no changes in the percentage of MCS were observed when StARD3 was depleted, despite the transfer of cholesterol to lipid droplets being completely blocked under these conditions. This strongly indicates that tethering and cholesterol transport at the MCS interface are two independent functions. Indeed, StARD3-depleted CHO M12-A6ko cells, showed no differences in MCS numbers (compared with CHO M12-A6ko), though no lipid droplets were observed (Fig. 5a, S4c).

## Discussion

We have shown here that the loss of NPC1 function and the concomitant accumulation of cholesterol in the late endocytic compartment can be rescued by AnxA6 depletion. We found that AnxA6 interacts with TBC1D15 (Rab7-GAP), enabling TBC1D15 to inactivate Rab7. Consequently, AnxA6 as well as TBC1D15 depletion lead to elevated Rab7-GTP levels, which facilitates late endosome-cholesterol egress, increased late endosome motility and a concurrent increase of neutral lipid accumulation in lipid droplets, in an ACAT-dependent manner. Mechanistically, we showed that StARD3 is instrumental for the transfer of late endosome-cholesterol to the ER in NPC1 mutants lacking AnxA6.

**Fig. 6:**
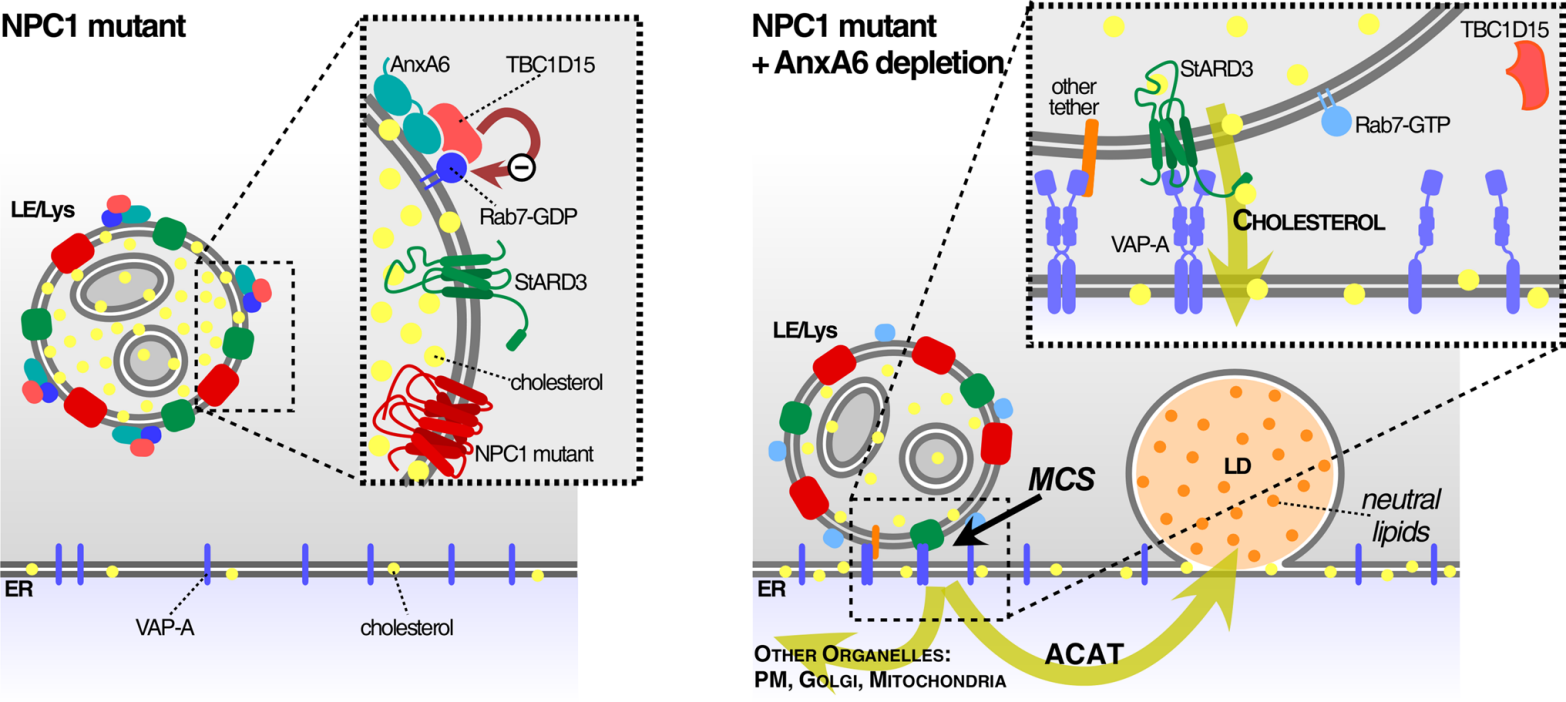
Model. Scheme of the proposed mechanism for AnxA6 in Rab7 inactivation and membrane contact site (MCS) functioning. Increased amounts of AnxA6 in cholesterol-laden late endosomes (LE) of NPC1 mutant cells enables the recruitment of the Rab7-GAP, TBC1D15, which inactivates Rab7. Lowering AnxA6 levels in late endosomes of NPC1 mutant cells leads to elevated amounts of Rab7-GTP and upregulation of StARD3. This facilitates the formation of MCS to establish LDL-cholesterol transfer to the ER, followed by cholesterol esterification in an ACAT-dependent manner and cholesteryl-ester storage (neutral lipids) in lipid droplets (LDs). StARD3/VAP-A seems to be instrumental for the cholesterol transfer from late endosomes to ER through MCS. This concomitantly reduces late endosome-cholesterol accumulation in NPC1 mutant cells. Most likely other tethers also operate at this interface, since StARD3 depletion interferes with cholesterol transfer from LE to LD yet MCS formation was not affected (see text for details)

Therefore, AnxA6 is a novel component of the cellular machinery regulating cellular cholesterol homeostasis (Fig. 6). Late endosome-cholesterol accumulation in NPC1 mutant cells is associated with elevated AnxA6 protein levels, which is detrimental for the trafficking and dynamics of the late endocytic compartment because it blocks Rab7 activation. Increased AnxA6 levels in NPC1 mutant CHO cell lines shown here, human NPC1 mutant skin fibroblasts (GM03123, data not shown), and U18666A-treated CHO-WT cells [44] can possibly be explained by a KFERQ-motif in AnxA6, which targets AnxA6 via chaperone-mediated autophagy (CMA) or endosomal microautophagy [81–83]. However, CMA is markedly inhibited upon late endosome-cholesterol accumulation [83–88], which might explain hampered CMA-mediated AnxA6 degradation.

On the other hand, it is now well established that cholesterol levels in endolysosomes can regulate the positioning of this organelle. A sophisticated ensemble of tethers, OSBPs, motor proteins and components of the cytoskeleton accomplishes the spatio-temporal re-organization of late endosomes and lysosomes between the perinuclear area to the cell periphery [15, 16, 77]. The currently best understood mechanism involves cholesterol driving ORP1L interaction with Rab7 and phosphoinositides to facilitate minus-end transport, leading to enlarged, cholesterol-rich late endosome/lysosome vesicles in the perinuclear region. In contrast, when cholesterol levels are low, ORP1L undergoes a conformational change that allows interaction with VAP proteins in the ER and MCS formation in the cell periphery, permitting cholesterol transfer between these two compartments [27, 78, 89–91]. Hence, in NPC1 mutant cells late endosome-cholesterol accumulation is responsible for late endosome/lysosome clustering and transport collapse at the minus-end [27]. Although ORP1L and Rab7 are the main drivers in this process, NPC1 activity is required for ORP1L function [78]. These findings are based on studies in MelJuSo [27], HeLa [78, 90, 91] and A549 [92] cells, yet NPC1 mutant CHO cell lines examined in the current study do not express significant amounts of ORP1L. Hence other proteins and mechanisms that allow for MCS formation and cholesterol transfer, including StARD3, need to be considered.

The involvement of MCS in Rab7-dependent late endosome functionality was strongly supported by the increased contact surface between the ER and late endosomes. StARD3 depletion blocking late endosome-cholesterol export was in line with neutral lipid deposition in lipid droplets of CHO M12-A6ko cells. These findings are consistent with StARD3 upregulation in NPC1 mutant CHO cells and livers of NPC1 KO-mice [93] and StARD3 overexpression inducing MCS formation between late endosomes and the ER [26].

Although a number of MCS constituents between late endosomes and the ER have been identified (for a recent review see: [20]), the understanding of their regulation and dynamics remains elusive, and additional tethering/scaffolding proteins, such as MOSPD2 [75], AnxA1 [90], VPS13 proteins [94], Gramd1b [39] or lipids (PtdIns) have also been implicated [10]. With regard to cholesterol homeostasis, unsolved mechanisms include the kinetics and directionality of cholesterol transfer. For example, in HeLa cells the StARD3/VAP-A complex mediates cholesterol transport from the ER to late endosomes independently of ORP1L [75, 76]; however, using the same protein machinery, transport of cholesterol from late endosomes to the ER was also demonstrated [92]. Based on our findings, one can envisage that late endosome-cholesterol accumulation caused by loss of NPC1 would trigger an elevation of AnxA6 levels, possibly due to inhibition of CMA [83], and a concomitant increased recruitment of AnxA6 to late endosomes [43–45]. In CHO M12 cells, this enlarged pool of AnxA6 proteins could further potentiate late endosome-cholesterol accumulation and possibly impair their ability to fuse with other structures, for example autophagosomes or phagosomes. In line with this model, active Rab7-GTP was not detected on phagosomes from cholesterol-laden cells [95]. This would not only result in an increased ability of AnxA6/TBC1D15 to inactivate Rab7, but would also interfere with StARD3-dependent MCS formation/functioning, thereby inhibiting alternative late endosome-cholesterol export routes in NPC1 mutant cells.

Active GTP-bound lysosomal Rab7 is also involved in MCS formation via its direct interaction with protrudin [9–12, 18, 19]. This points to dual mechanistic tasks for activated Rab7 after AnxA6 depletion in NPC1 mutant cells: (i) to confer increased late endosome motility and cholesterol transport and (ii) to stabilize MCS through Rab7/protrudin/VAP-A complex formation. This implicates that cholesterol transport, which is inhibited by StARD3 depletion in AnxA6-depleted M12 cells, and MCS formation, which is not inhibited in these settings, are regulated separately. Depletion of AnxA6 could stabilize StARD3/VAP-A and Rab7/protrudin/VAP-A complexes, ensuring re-establishment of MCS between the ER and late endosomes, and eventually the transfer of late endosome-cholesterol to the ER in cells with non-functional NPC1. Although several annexins contribute to endosomal membrane dynamics [62, 96, 97], only the AnxA1/S100A11 protein complex has yet been associated with MCS formation [90]. While AnxA2 and AnxA6 are also well known to bind S100 proteins, to our knowledge there is no data linking AnxA2 with MCS [90, 98]. On the other hand, in the present study, the presence of AnxA6 seems to confer untethering of MCS between LE/Lys and ER in NPC1 mutant cells. Indeed, recent findings support that untethering of mitochondria–lysosome contacts is mediated by the recruitment of TBC1D15 to elicit Rab7-GTP hydrolysis and thereby release contacts [18, 19]. Alternatively, AnxA6 may interact with Rab7-GTP similar to ORP1L, which binds Rab7 via its ANK domain, excluding a direct effect on Rab7 GTPase activity [99].

We propose a model (Fig. 6) in which lowering AnxA6 levels on late endosomes of NPC1 mutant cells, characterized by upregulated StARD3 expression [93], leads to elevated Rab7-GTP levels. This enables the formation of MCS to establish LDL-cholesterol transfer to the ER, followed by increased cholesterol delivery to lipid droplets, ultimately decreasing late endosome-cholesterol accumulation in NPC1 mutant cells.

## Electronic supplementary material

Below is the link to the electronic supplementary material.

Supplementary material 1 (DOCX 66 kb)

Characterization of AnxA6/TBC1D15 interaction. (A) Representative images of CHO M12 and CHO M12-A6ko cells expressing GFP-Rab7-Q67L (green) and YFP-TBC1D15 (red). Areas of interest are shown at higher magnification. White arrowheads point at GFP-Rab7-Q67L positive vesicles. Line profile of fluorescence intensities of GFP-Rab7-Q67L (green) and YFP-TBC1D15 (red) are shown (A-B and C-D, 3-4 μm). Scale bar, 10 and 5 μm. Quantification shows the relative number of late endosomes with GFP/YFP (n=15 cells). (B) Subcellular fractionation of cell lysates from CHO-WT and CHO-A6 cells on discontinuous sucrose gradients. Fractions (F1-F2) were collected from top, separated by gel electrophoresis and immunoblotted for TBC1D15 and Rab7 as indicated. Cell lysates (5% of total input) are shown. Relative protein levels of TBC1D15 in the Rab7-positive late endosomal fraction (F2) were normalized to total TBC1D15 levels (see input) and are shown in the right panel (n=3). (C) Representative electron micrograph of the late endosome/lysosome (LE/Lys) fraction F2 from sucrose gradients of CHO cells (Fig. S2B). Prototypical endolysosomal structures with internal membranes can be observed (*). Insert shows a high magnification of a multilamellar structure. Scale bar: 500 and 200 nm. ** p<0.01 by two-tailed Student’s t-test (A and B). Data is shown as mean ± SEM (TIFF 2503 kb)

Regulation of Rab7 activity and late endosome-cholesterol egress. Total levels of Rab7, AnxA6 and actin in cell lysates (5% of total input) and the quantification of relative Rab7 activity are shown (n=3). Rab7-GTP levels determined as in Fig. 2F-2H with cell lysates from (A) A431-WT and A431-A6, or (B) mouse embryonic fibroblasts from wildtype (WT) and AnxA6-KO (A6ko) mice. (C-D) CHO M12 or CHO M12-A6ko cells were transfected with empty vector (GFP), GFP-Rab7-Q67L, YFPTBC1D15( 1-200) or GFP-Rab7-T22N (green) as indicated, fixed and stained with filipin (red). For better comparison of filipin staining, the outline and shape of cells is indicated (transfected cells in yellow). Merged images are shown. Scale bar, 10 μm. The mean relative filipin intensity of at least 20 transfected vs. non-transfected cells was quantified (n=3). (E) CHO M12 cells expressing control siRNA (siCtrl) or siRNA targeting TBC1D15 (siTBC1D15) were starved in 5% LPDS for 48 h and loaded with 50 μg/ml LDL for 24 h as above. Then cells were fixed, stained with filipin (cholesterol, red) and BODIPY 493/503 (neutral lipids, green), and representative fields (merged and split channels) are shown. Enlarged regions of interest are shown. For better comparison of filipin and BODIPY staining, the outline and shape of cells is indicated. Scale bar, 10 μm. (F) Representative western blot and quantification (normalized to actin) showing siRNA-mediated TBC1D15 depletion in CHO M12 cells (n=3). (G-H) Dot-plot of number, area and relative intensity of filipin-stained (late endosomes) and BODIPY-stained (lipid droplets) vesicles per cell of a representative experiment (n > 60, 3 experiments). For quantification details see Methods. ** p<0.01; *** p<0.001 by two-tailed Student’s t-test (A, B, C, D, F, G, H). Data are presented as mean ± SEM (A, B, C, D, F) and mean ± SD in red (G, H) (TIFF 3757 kb)

Delipidation and LDL-loading experiment procedure. (A) Scheme of experimental protocol for delipidation and LDL loading, and AnxA6 siRNA depletion control in CHO M12 cells. (B) CHO-WT and CHO M12 cells were grown in 10% FCS (0 h, control), then starved in 5% LPDS for 48 h before loading with 50 μg/ml LDL for 24 h. At each time point (0, 48 and 72 h), cells were fixed, stained with filipin (cholesterol, red) and BODIPY 493/503 (neutral lipids, green). Representative fields of cells at t=0 (control), t=48 (LPDS) and t=72 h (LDL) are shown (merged and split channels). Enlarged regions of interest are shown. For better comparison of filipin and BODIPY staining, the outline and shape of cells is indicated. Scale bar, 10 μm (TIFF 2904 kb)

Characterization of neutral lipid and cholesterol distribution in CHO M12 and CHO M12-A6ko cells. (A) CHO M12 and CHO M12-A6ko cells were grown under normal conditions. Cells were fixed, immunolabelled with the lipid droplet marker anti-adipophilin (red) and stained with filipin (blue) and BODIPY (green) as indicated. Representative images and quantification of adipophilinpositive vesicles and filipin intensity per cell (n > 20 cells, 2 experiments) are shown. For quantification details see Methods. White squares outline enlarged inserts (1-2). Scale bar, 10 μm. (B) CHO M12-A6ko cells were starved in 5% LPDS for 48 h before loading with 50 μg/ml LDL for 24 h fixed, immunolabeled with anti-adipophilin (red) and stained with filipin (blue) and BODIPY (green). Separate and merged channels are shown. Arrowheads point at representative BODIPY- and adipophilin-positive lipid droplets in the perinuclear region. Scale bar, 10 μm. (C) Conventional transmission electron microscopy (TEM) showing representative images and quantitation of lipid droplets (red asterisks) and MCS in CHO-WT, CHO M12, CHO M12-A6ko and StARD3-depleted CHO M12-A6ko (CHO M12-A6ko siRNA-StARD3) cells loaded with LDL for 24 h as indicated (see details in Methods) (D). Abundant lipid droplets, as characterized by translucent electron density, can be observed in CHO-WT and CHO M12-A6ko cells. Note the close contacts between lipid droplets and late endosomes/lysosomes (LE/Lys) structures in CHO M12-A6ko cells (red squares). Lipid droplets with an electron-dense ‘‘cap’’ (white arrow) of lipofuscinlike structures possibly pointing at early steps to initiate the sequestration/engulfing portions of LD for degradation. Mit, mitochondria; ER, endoplasmic reticulum; Nuc, nucleus; PM, plasma membrane. Scale bar, 200 nm. *** p<0.001 by two-tailed Student’s t-test (A) or one-way ANOVA with Bonferroni post-hoc test (C). All data is presented as mean ± SD in red (TIFF 8419 kb)

Reduced expression of mature SREBP2 (mSREBP2) in AnxA6-depleted CHO M12 cells. CHO M12 expressing control siRNA (siRNA Ctrl) or siRNA targeting AnxA6 (siRNA AnxA6) were grown in 5% LPDS and 10 mM mevastatin for 3 days before loading with 50 μg/ml LDL for 0, 4 and 8 h as indicated. Whole cell lysates were prepared at each time point and analyzed by Western blotting for mature SREBP2 (mSREBP2), glyceraldehyde 3-phosphate dehydrogenase (GAPDH) and AnxA6. Relative mSREBP2 levels were quantified and normalized to GAPDH. AnxA6 depletion results in less mSREBP2 protein expression (~ 20%) after 8 h LDL loading. A representative Western blot and quantification from 2 independent experiments with duplicate samples is shown. Data is presented as mean ± SEM (TIFF 378 kb)

Lack of ORP1L expression in CHO cells. RNA samples from CHO cells transfected ± ORP1L-GFP (mouse) were analyzed by RT-PCR for the expression of endogenous (hamster ORP1L) and transfected ORP1L-GFP (primer sequence from homologous hamster and mouse regions). The housekeeper hamster gene RPL13 served as control. While transfected ORP1L-GFP was readily detectable (mean Cq 18.37), hamster ORP1L could only be detected after more than 30 PCR cycles (mean Cq 32.77) indicating very low expression of ORP1L in CHO cells (TIFF 107 kb)

Motility of BODIPY-Cholesterol-labelled late endosomes in control siRNA-transfected CHO M12 cells (CHO M12 siCtrl) 24 h after pulse-labelling with LDL-BODIPY-cholesteryl linoleate. Confocal timelapse images were captured with image acquisition frame rate of 370 msec (MP4 1539 kb)

Motility of BODIPY-Cholesterol-labelled late endosomes in AnxA6 siRNA-transfected CHO M12 cells (CHO M12 siAnxA6) 24 h after pulse-labelling with LDL-BODIPY-cholesteryl linoleate. Confocal timelapse images were captured with image acquisition frame rate of 370 msec (MP4 1682 kb)

## Abbreviations

A431: Human epidermoid carcinoma cells
ACAT: Acyl-CoA:cholesterol acyltransferase
AnxA6: Annexin A6
CHO: Chinese hamster ovary
CHO M12: NPC1 mutant CHO cell line
CMA: Chaperone-mediated autophagy
ER: Endoplasmic reticulum
FYCO1: FYVE and coiled-coil domain containing 1
GST: Glutathione S-transferase
LE/Lys: Late endosome/lysosome (endolysosomes)
LPDS: Lipoprotein-deficient serum
MCS: Membrane contact sites
MEF: Mouse embryonic fibroblasts
MOSPD2: Motile sperm domain containing 2
NPC1: Niemann-Pick type C1
ORP1L: Oxysterol-related protein 1L
OSBP: Oxysterol-binding protein
PFO: Perfringolysin O
RILP: Rab interacting lysosomal protein
SREBP: Sterol regulatory element binding protein
StARD3: StAR-related lipid transfer domain-3
TBC1D15: TBC1 domain family member 15
WT: Wild type
VAP-A: Vesicle-associated membrane protein-associated protein A
Vps13: Vacuolar protein sorting-associated protein 13

## References

[1] Urano Y, Watanabe H, Murphy SR, Shibuya Y, Geng Y, Peden AA, Chang CC, Chang TY (2008). Transport of LDL-derived cholesterol from the NPC1 compartment to the ER involves the trans-Golgi network and the SNARE protein complex. Proc Natl Acad Sci USA.

[2] Mesmin B, Maxfield FR (2009). Intracellular sterol dynamics. Biochim Biophys Acta.

[3] Ikonen E (2008). Cellular cholesterol trafficking and compartmentalization. Nat Rev Mol Cell Biol.

[4] Ikonen E (2018). Mechanisms of cellular cholesterol compartmentalization: recent insights. Curr Opin Cell Biol.

[5] Gomez NM, Lu W, Lim JC, Kiselyov K, Campagno KE, Grishchuk Y, Slaugenhaupt SA, Pfeffer BA, Fliesler SJ, Mitchell CH (2018). Robust lysosomal calcium signaling through channel TRPML1 is impaired by lysosomal lipid accumulation. FASEB J.

[6] Luo J, Jiang L, Yang H, Song BL (2017). Routes and mechanisms of post-endosomal cholesterol trafficking: a story that never ends. Traffic.

[7] Levine T (2004). Short-range intracellular trafficking of small molecules across endoplasmic reticulum junctions. Trends Cell Biol.

[8] Phillips MJ, Voeltz GK (2016). Structure and function of ER membrane contact sites with other organelles. Nat Rev Mol Cell Biol.

[9] Raiborg C, Wenzel EM, Pedersen NM, Olsvik H, Schink KO, Schultz SW, Vietri M, Nisi V, Bucci C, Brech A, Johansen T, Stenmark H (2015). Repeated ER-endosome contacts promote endosome translocation and neurite outgrowth. Nature.

[10] Raiborg C, Wenzel EM, Pedersen NM, Stenmark H (2016). Phosphoinositides in membrane contact sites. Biochem Soc Trans.

[11] Raiborg C, Wenzel EM, Stenmark H (2015). ER-endosome contact sites: molecular compositions and functions. EMBO J.

[12] Wijdeven RH, Jongsma ML, Neefjes J, Berlin I (2015). ER contact sites direct late endosome transport. BioEssays.

[13] van der Kant R, Neefjes J (2014). Small regulators, major consequences - Ca(2)(+) and cholesterol at the endosome-ER interface. J Cell Sci.

[14] Pfisterer SG, Peranen J, Ikonen E (2016). LDL-cholesterol transport to the endoplasmic reticulum: current concepts. Curr Opin Lipidol.

[15] Luo J, Jiang LY, Yang H, Song BL (2018). Intracellular cholesterol transport by sterol transfer proteins at membrane contact sites. Trends Biochem Sci.

[16] Wu H, Carvalho P, Voeltz GK (2018). Here, there, and everywhere: the importance of ER membrane contact sites. Science.

[17] Ridgway ND, Zhao K (2018). Cholesterol transfer at endosomal-organelle membrane contact sites. Curr Opin Lipidol.

[18] Wong LH, Eden ER, Futter CE (2018). Roles for ER:endosome membrane contact sites in ligand-stimulated intraluminal vesicle formation. Biochem Soc Trans.

[19] Wong YC, Ysselstein D, Krainc D (2018). Mitochondria-lysosome contacts regulate mitochondrial fission via RAB7 GTP hydrolysis. Nature.

[20] Scorrano L, De Matteis MA, Emr S, Giordano F, Hajnoczky G, Kornmann B, Lackner LL, Levine TP, Pellegrini L, Reinisch K, Rizzuto R, Simmen T, Stenmark H, Ungermann C, Schuldiner M (2019). Coming together to define membrane contact sites. Nat Commun.

[21] Hulce JJ, Cognetta AB, Niphakis MJ, Tully SE, Cravatt BF (2013). Proteome-wide mapping of cholesterol-interacting proteins in mammalian cells. Nat Methods.

[22] Alpy F, Latchumanan VK, Kedinger V, Janoshazi A, Thiele C, Wendling C, Rio MC, Tomasetto C (2005). Functional characterization of the MENTAL domain. J Biol Chem.

[23] Holtta-Vuori M, Alpy F, Tanhuanpaa K, Jokitalo E, Mutka AL, Ikonen E (2005). MLN64 is involved in actin-mediated dynamics of late endocytic organelles. Mol Biol Cell.

[24] Alpy F, Stoeckel ME, Dierich A, Escola JM, Wendling C, Chenard MP, Vanier MT, Gruenberg J, Tomasetto C, Rio MC (2001). The steroidogenic acute regulatory protein homolog MLN64, a late endosomal cholesterol-binding protein. J Biol Chem.

[25] van der Kant R, Zondervan I, Janssen L, Neefjes J (2013). Cholesterol-binding molecules MLN64 and ORP1L mark distinct late endosomes with transporters ABCA3 and NPC1. J Lipid Res.

[26] Alpy F, Rousseau A, Schwab Y, Legueux F, Stoll I, Wendling C, Spiegelhalter C, Kessler P, Mathelin C, Rio MC, Levine TP, Tomasetto C (2013). STARD3 or STARD3NL and VAP form a novel molecular tether between late endosomes and the ER. J Cell Sci.

[27] Rocha N, Kuijl C, van der Kant R, Janssen L, Houben D, Janssen H, Zwart W, Neefjes J (2009). Cholesterol sensor ORP1L contacts the ER protein VAP to control Rab7-RILP-p150 Glued and late endosome positioning. J Cell Biol.

[28] Charman M, Kennedy BE, Osborne N, Karten B (2010). MLN64 mediates egress of cholesterol from endosomes to mitochondria in the absence of functional Niemann–Pick Type C1 protein. J Lipid Res.

[29] Elustondo P, Martin LA, Karten B (2017). Mitochondrial cholesterol import. Biochim Biophys Acta.

[30] Vassilev B, Sihto H, Li S, Holtta-Vuori M, Ilola J, Lundin J, Isola J, Kellokumpu-Lehtinen PL, Joensuu H, Ikonen E (2015). Elevated levels of StAR-related lipid transfer protein 3 alter cholesterol balance and adhesiveness of breast cancer cells: potential mechanisms contributing to progression of HER2-positive breast cancers. Am J Pathol.

[31] Borthwick F, Allen AM, Taylor JM, Graham A (2010). Overexpression of STARD3 in human monocyte/macrophages induces an anti-atherogenic lipid phenotype. Clin Sci (Lond).

[32] Liapis A, Chen FW, Davies JP, Wang R, Ioannou YA (2012). MLN64 transport to the late endosome is regulated by binding to 14-3-3 via a non-canonical binding site. PLoS One.

[33] Bucci C, Thomsen P, Nicoziani P, McCarthy J, van Deurs B (2000). Rab7: a key to lysosome biogenesis. Mol Biol Cell.

[34] Ganley IG, Wong PM, Gammoh N, Jiang X (2011). Distinct autophagosomal-lysosomal fusion mechanism revealed by thapsigargin-induced autophagy arrest. Mol Cell.

[35] Lebrand C, Corti M, Goodson H, Cosson P, Cavalli V, Mayran N, Faure J, Gruenberg J (2002). Late endosome motility depends on lipids via the small GTPase Rab7. EMBO J.

[36] Choudhury A, Dominguez M, Puri V, Sharma DK, Narita K, Wheatley CL, Marks DL, Pagano RE (2002). Rab proteins mediate Golgi transport of caveola-internalized glycosphingolipids and correct lipid trafficking in Niemann–Pick C cells. J Clin Invest.

[37] Linder MD, Uronen RL, Holtta-Vuori M, van der Sluijs P, Peranen J, Ikonen E (2007). Rab8-dependent recycling promotes endosomal cholesterol removal in normal and sphingolipidosis cells. Mol Biol Cell.

[38] Cianciola NL, Greene DJ, Morton RE, Carlin CR (2013). Adenovirus RIDalpha uncovers a novel pathway requiring ORP1L for lipid droplet formation independent of NPC1. Mol Biol Cell.

[39] Hoglinger D, Burgoyne T, Sanchez-Heras E, Hartwig P, Colaco A, Newton J, Futter CE, Spiegel S, Platt FM, Eden ER (2019). NPC1 regulates ER contacts with endocytic organelles to mediate cholesterol egress. Nat Commun.

[40] Garcia-Melero A, Reverter M, Hoque M, Meneses-Salas E, Koese M, Conway JR, Johnsen CH, Alvarez-Guaita A, Morales-Paytuvi F, Elmaghrabi YA, Pol A, Tebar F, Murray RZ, Timpson P, Enrich C, Grewal T, Rentero C (2016). Annexin A6 and late endosomal cholesterol modulate integrin recycling and cell migration. J Biol Chem.

[41] Enrich C, Rentero C, de Muga SV, Reverter M, Mulay V, Wood P, Koese M, Grewal T (2011). Annexin A6-linking Ca(2+) signaling with cholesterol transport. Biochim Biophys Acta.

[42] Gerke V, Creutz CE, Moss SE (2005). Annexins: linking Ca2+ signalling to membrane dynamics. Nat Rev Mol Cell Biol.

[43] Grewal T, Heeren J, Mewawala D, Schnitgerhans T, Wendt D, Salomon G, Enrich C, Beisiegel U, Jackle S (2000). Annexin VI stimulates endocytosis and is involved in the trafficking of low density lipoprotein to the prelysosomal compartment. J Biol Chem.

[44] de Diego I, Schwartz F, Siegfried H, Dauterstedt P, Heeren J, Beisiegel U, Enrich C, Grewal T (2002). Cholesterol modulates the membrane binding and intracellular distribution of annexin 6. J Biol Chem.

[45] te Vruchte D, Lloyd-Evans E, Veldman RJ, Neville DC, Dwek RA, Platt FM, van Blitterswijk WJ, Sillence DJ (2004). Accumulation of glycosphingolipids in Niemann-Pick C disease disrupts endosomal transport. J Biol Chem.

[46] Cubells L, Vila de Muga S, Tebar F, Wood P, Evans R, Ingelmo-Torres M, Calvo M, Gaus K, Pol A, Grewal T, Enrich C (2007). Annexin A6-induced alterations in cholesterol transport and caveolin export from the Golgi complex. Traffic.

[47] Havel RJ, Eder HA, Bragdon JH (1955). The distribution and chemical composition of ultracentrifugally separated lipoproteins in human serum. J Clin Invest.

[48] Goldstein JL, Basu SK, Brown MS (1983). Receptor-mediated endocytosis of low-density lipoprotein in cultured cells. Methods Enzymol.

[49] Cubells L, Vila de Muga S, Tebar F, Bonventre JV, Balsinde J, Pol A, Grewal T, Enrich C (2008). Annexin A6-induced inhibition of cytoplasmic phospholipase A2 is linked to caveolin-1 export from the Golgi. J Biol Chem.

[50] Grewal T, Evans R, Rentero C, Tebar F, Cubells L, de Diego I, Kirchhoff MF, Hughes WE, Heeren J, Rye KA, Rinninger F, Daly RJ, Pol A, Enrich C (2005). Annexin A6 stimulates the membrane recruitment of p120GAP to modulate Ras and Raf-1 activity. Oncogene.

[51] Alvarez-Guaita A, Vila de Muga S, Owen DM, Williamson D, Magenau A, Garcia-Melero A, Reverter M, Hoque M, Cairns R, Cornely R, Tebar F, Grewal T, Gaus K, Ayala-Sanmartin J, Enrich C, Rentero C (2015). Evidence for annexin A6-dependent plasma membrane remodelling of lipid domains. Br J Pharmacol.

[52] Ran FA, Hsu PD, Wright J, Agarwala V, Scott DA, Zhang F (2013). Genome engineering using the CRISPR-Cas9 system. Nat Protoc.

[53] Schneider CA, Rasband WS, Eliceiri KW (2012). NIH Image to ImageJ: 25 years of image analysis. Nat Methods.

[54] Pons M, Ihrke G, Koch S, Biermer M, Pol A, Grewal T, Jackle S, Enrich C (2000). Late endocytic compartments are major sites of annexin VI localization in NRK fibroblasts and polarized WIF-B hepatoma cells. Exp Cell Res.

[55] Kanerva K, Uronen RL, Blom T, Li S, Bittman R, Lappalainen P, Peranen J, Raposo G, Ikonen E (2013). LDL cholesterol recycles to the plasma membrane via a Rab8a-Myosin5b-actin-dependent membrane transport route. Dev Cell.

[56] Sun Q, Westphal W, Wong KN, Tan I, Zhong Q (2010). Rubicon controls endosome maturation as a Rab7 effector. Proc Natl Acad Sci USA.

[57] Itoh RE, Kurokawa K, Fujioka A, Sharma A, Mayer BJ, Matsuda M (2005). A FRET-based probe for epidermal growth factor receptor bound non-covalently to a pair of synthetic amphipathic helixes. Exp Cell Res.

[58] Yamano K, Fogel AI, Wang C, van der Bliek AM, Youle RJ (2014). Mitochondrial Rab GAPs govern autophagosome biogenesis during mitophagy. Elife.

[59] Cantalupo G, Alifano P, Roberti V, Bruni CB, Bucci C (2001). Rab-interacting lysosomal protein (RILP): the Rab7 effector required for transport to lysosomes. EMBO J.

[60] Das A, Goldstein JL, Anderson DD, Brown MS, Radhakrishnan A (2013). Use of mutant 125I-perfringolysin O to probe transport and organization of cholesterol in membranes of animal cells. Proc Natl Acad Sci USA.

[61] Ollion J, Cochennec J, Loll F, Escude C, Boudier T (2013). TANGO: a generic tool for high-throughput 3D image analysis for studying nuclear organization. Bioinformatics.

[62] Rentero C, Blanco-Munoz P, Meneses-Salas E, Grewal T, Enrich C (2018). Annexins-coordinators of cholesterol homeostasis in endocytic pathways. Int J Mol Sci.

[63] Kwiatkowska K, Marszalek-Sadowska E, Traczyk G, Koprowski P, Musielak M, Lugowska A, Kulma M, Grzelczyk A, Sobota A (2014). Visualization of cholesterol deposits in lysosomes of Niemann-Pick type C fibroblasts using recombinant perfringolysin O. Orphanet J Rare Dis.

[64] Peralta ER, Martin BC, Edinger AL (2010). Differential effects of TBC1D15 and mammalian Vps39 on Rab7 activation state, lysosomal morphology, and growth factor dependence. J Biol Chem.

[65] Chen YN, Gu X, Zhou XE, Wang W, Cheng D, Ge Y, Ye F, Xu HE, Lv Z (2017). Crystal structure of TBC1D15 GTPase-activating protein (GAP) domain and its activity on Rab GTPases. Protein Sci.

[66] Frasa MA, Koessmeier KT, Ahmadian MR, Braga VM (2012). Illuminating the functional and structural repertoire of human TBC/RABGAPs. Nat Rev Mol Cell Biol.

[67] Fukuda M (2011). TBC proteins: Gaps for mammalian small GTPase Rab?. Biosci Rep.

[68] Nottingham RM, Pfeffer SR (2009). Defining the boundaries: rab GEFs and GAPs. Proc Natl Acad Sci USA.

[69] Zhang XM, Walsh B, Mitchell CA, Rowe T (2005). TBC domain family, member 15 is a novel mammalian Rab GTPase-activating protein with substrate preference for Rab7. Biochem Biophys Res Commun.

[70] Rentero C, Evans R, Wood P, Tebar F, Vila de Muga S, Cubells L, de Diego I, Hayes TE, Hughes WE, Pol A, Rye KA, Enrich C, Grewal T (2006). Inhibition of H-Ras and MAPK is compensated by PKC-dependent pathways in annexin A6 expressing cells. Cell Signal.

[71] Heid HW, Moll R, Schwetlick I, Rackwitz HR, Keenan TW (1998). Adipophilin is a specific marker of lipid accumulation in diverse cell types and diseases. Cell Tissue Res.

[72] Brown MS, Radhakrishnan A, Goldstein JL (2017). Retrospective on cholesterol homeostasis: the central role of scap. Annu Rev Biochem.

[73] Kristiana I, Yang H, Brown AJ (2008). Different kinetics of cholesterol delivery to components of the cholesterol homeostatic machinery: implications for cholesterol trafficking to the endoplasmic reticulum. Biochim Biophys Acta.

[74] Ross AC, Go KJ, Heider JG, Rothblat GH (1984). Selective inhibition of acyl coenzyme A:cholesterol acyltransferase by compound 58-035. J Biol Chem.

[75] Di Mattia T, Wilhelm LP, Ikhlef S, Wendling C, Spehner D, Nomine Y, Giordano F, Mathelin C, Drin G, Tomasetto C, Alpy F (2018). Identification of MOSPD2, a novel scaffold for endoplasmic reticulum membrane contact sites. EMBO Rep..

[76] Wilhelm LP, Wendling C, Vedie B, Kobayashi T, Chenard MP, Tomasetto C, Drin G, Alpy F (2017). STARD3 mediates endoplasmic reticulum-to-endosome cholesterol transport at membrane contact sites. EMBO J.

[77] Cabukusta B, Neefjes J (2018). Mechanisms of lysosomal positioning and movement. Traffic.

[78] Zhao K, Ridgway ND (2017). Oxysterol-binding protein-related protein 1l regulates cholesterol egress from the endo-lysosomal system. Cell Rep.

[79] Du X, Kumar J, Ferguson C, Schulz TA, Ong YS, Hong W, Prinz WA, Parton RG, Brown AJ, Yang H (2011). A role for oxysterol-binding protein-related protein 5 in endosomal cholesterol trafficking. J Cell Biol.

[80] Vacca F, Scott C, Gruenberg J (2016). The Late Endosome. Encycl Cell Biol.

[81] Cuervo AM, Gomes AV, Barnes JA, Dice JF (2000). Selective degradation of annexins by chaperone-mediated autophagy. J Biol Chem.

[82] Kaushik S, Cuervo AM (2012). Chaperone-mediated autophagy: a unique way to enter the lysosome world. Trends Cell Biol.

[83] Rodriguez-Navarro JA, Kaushik S, Koga H, Dall’Armi C, Shui G, Wenk MR, Di Paolo G, Cuervo AM (2012). Inhibitory effect of dietary lipids on chaperone-mediated autophagy. Proc Natl Acad Sci USA.

[84] Sobo K, Le Blanc I, Luyet PP, Fivaz M, Ferguson C, Parton RG, Gruenberg J, van der Goot FG (2007). Late endosomal cholesterol accumulation leads to impaired intra-endosomal trafficking. PLoS One.

[85] Fraldi A, Annunziata F, Lombardi A, Kaiser HJ, Medina DL, Spampanato C, Fedele AO, Polishchuk R, Sorrentino NC, Simons K, Ballabio A (2010). Lysosomal fusion and SNARE function are impaired by cholesterol accumulation in lysosomal storage disorders. EMBO J.

[86] Koga H, Kaushik S, Cuervo AM (2010). Altered lipid content inhibits autophagic vesicular fusion. FASEB J.

[87] Castellano BM, Thelen AM, Moldavski O, Feltes M, van der Welle RE, Mydock-McGrane L, Jiang X, van Eijkeren RJ, Davis OB, Louie SM, Perera RM, Covey DF, Nomura DK, Ory DS, Zoncu R (2017). Lysosomal cholesterol activates mTORC1 via an SLC38A9-Niemann-Pick C1 signaling complex. Science.

[88] Tsuji T, Fujimoto M, Tatematsu T, Cheng J, Orii M, Takatori S, Fujimoto T (2017). Niemann-Pick type C proteins promote microautophagy by expanding raft-like membrane domains in the yeast vacuole. Elife.

[89] Johansson M, Rocha N, Zwart W, Jordens I, Janssen L, Kuijl C, Olkkonen VM, Neefjes J (2007). Activation of endosomal dynein motors by stepwise assembly of Rab7-RILP-p150Glued, ORP1L, and the receptor betalll spectrin. J Cell Biol.

[90] Eden ER, Sanchez-Heras E, Tsapara A, Sobota A, Levine TP, Futter CE (2016). Annexin A1 Tethers membrane contact sites that mediate ER to endosome cholesterol transport. Dev Cell.

[91] Elgner F, Ren H, Medvedev R, Ploen D, Himmelsbach K, Boller K, Hildt E (2016). The intracellular cholesterol transport inhibitor U18666A inhibits the exosome-dependent release of mature hepatitis C virus. J Virol.

[92] Cianciola NL, Chung S, Manor D, Carlin CR (2017). Adenovirus modulates Toll-Like receptor 4 signaling by reprogramming ORP1L-VAP protein contacts for cholesterol transport from endosomes to the endoplasmic reticulum. J Virol.

[93] Balboa E, Castro J, Pinochet MJ, Cancino GI, Matias N, Jose Saez P, Martinez A, Alvarez AR, Garcia-Ruiz C, Fernandez-Checa JC, Zanlungo S (2017). MLN64 induces mitochondrial dysfunction associated with increased mitochondrial cholesterol content. Redox Biol.

[94] Kumar N, Leonzino M, Hancock-Cerutti W, Horenkamp FA, Li P, Lees JA, Wheeler H, Reinisch KM, De Camilli P (2018). VPS13A and VPS13C are lipid transport proteins differentially localized at ER contact sites. J Cell Biol.

[95] Huynh KK, Gershenzon E, Grinstein S (2008). Cholesterol accumulation by macrophages impairs phagosome maturation. J Biol Chem.

[96] Enrich C, Rentero C, Meneses-Salas E, Tebar F, Grewal T (2017). Annexins: Ca(2+) effectors determining membrane trafficking in the late endocytic compartment. Adv Exp Med Biol.

[97] Gruenberg J, Stenmark H (2004). The biogenesis of multivesicular endosomes. Nat Rev Mol Cell Biol.

[98] Rintala-Dempsey AC, Rezvanpour A, Shaw GS (2008). S100-annexin complexes–structural insights. FEBS J.

[99] Tong J, Tan L, Chun C, Im YJ (2019). Structural basis of human ORP1-Rab7 interaction for the late-endosome and lysosome targeting. PLoS One.

