# Supplementary material for: Annexin A6 modulates TBC1D15/Rab7/StARD3 axis to control endosomal cholesterol export in NPC1 cells
Source: Cell Mol Life Sci. 2019 Oct 29;77(14):2839–57. doi: 10.1007/s00018-019-03330-y (PMC7326902; doi:10.1007/s00018-019-03330-y)
Supplement: Supplementary file 1 — Supplementary material 1 (DOCX 66 kb) [file 18_2019_3330_MOESM1_ESM.docx]

**Annexin A6 modulates TBC1D15/Rab7/StARD3 axis to control endosomal cholesterol export in NPC1 cells**

**Elsa Meneses-Salas, Ana García-Melero, Kristiina Kanerva, Patricia Blanco-Muñoz, Frederic Morales-Paytuvi, Júlia Bonjoch, Josefina Casas, Antonia Egert, Syed S. Beevi, Jaimy Jose, Vicenta Llorente-Cortés, Kerry-Anne Rye, Joerg Heeren, Albert Lu, Albert Pol, Francesc Tebar, Elina Ikonen, Thomas Grewal, Carlos Enrich and Carles Rentero**

**SUPPLEMENTARY MATERIAL**

**Supplementary Table 1.** List of reagents used in this study.

| **REAGENT or RESOURCE** | **SOURCE** | **IDENTIFIER** |
| --- | --- | --- |
| **Antibodies** |  |  |
| Rabbit polyclonal anti-TBC1D15 | Abcam | ab-121396 |
| Rabbit polyclonal anti-AnxA6 | [[1](#_ENREF_1)] | N/A |
| Rabbit polyclonal anti-GFP | Abcam | ab290 |
| Rabbit polyclonal anti-Rab7 | Cell Signalling | 2094 |
| Mouse monoclonal anti-Actin | MP Biomedicals | 69.100 |
| Rabbit polyclonal anti-Adipophilin | Abcam | ab78920 |
| Rabbit polyclonal anti-GST | Abcam | ab19256 |
| Rabbit polyclonal anti-MLN64 | Abcam | ab3478 |
|  |  |  |
| **Secondary Antibodies** |  |  |
| Alexa Fluor 555 donkey anti-rabbit IgG | Invitrogen | A31572 |
| HRP goat anti-rabbit IgG | Bio-Rad | 170-6515 |
| HRP goat anti-mouse IgG | Bio-Rad | 170-6516 |
| HRP mouse anti-rabbit IgG light chain | Abcam | ab99697 |
|  |  |  |
| **Recombinant DNA** |  |  |
| pEYFP-C-TBC1D15 | generated from pEF6-myc-TBC1D15 [[2](#_ENREF_2)] | N/A |
| pEGFP-N1 | Clontech | 6085-1 |
| pEGFP-N1-AnxA6 | [[3](#_ENREF_3)] | N/A |
| pEGFP-C-Rab7-T22N | [[4](#_ENREF_4)] | Addgene #12660 |
| pcDNA3.1-GFP-Rab7-Q67L | [[5](#_ENREF_5)] | Addgene #28049 |
| RFP-Rab7 | [[6](#_ENREF_6)] | N/A |
| pEYFP-C-TBC1D15 [1-200] | [[7](#_ENREF_7)] | N/A |
| pEYFP-C-TBC1D15 [201-333] | [[7](#_ENREF_7)] | N/A |
| pEYFP-C-TBC1D15 [334-557] | [[7](#_ENREF_7)] | N/A |
| pEYFP-C-TBC1D15 [558-647] | [[7](#_ENREF_7)] | N/A |
| RILP-C33-GST | [[8](#_ENREF_8)] | N/A |
| pGEX-4T-AnxA6 | [[3](#_ENREF_3)] | N/A |
| pGEX-4T2-PFO (Y181A C459A) | [[9](#_ENREF_9)] | N/A |
| pSpCas9(BB)-2A-Puro v2 | [[10](#_ENREF_10)] | Addgene #62988 |
| pSpCas9(BB)-2A-Puro2-cAnxA6 #1 | This paper | N/A |
| pSpCas9(BB)-2A-Puro2-cAnxA6 #2 | This paper | N/A |
|  |  |  |
| **Experimental Models: Cell Lines** |  |  |
| CHO-WT (CHO-K1) | ECACC | 85051005 |
| CHO AnxA6 | [[3](#_ENREF_3)] | N/A |
| CHO M12 | Dr L Liscum | N/A |
| CHO 2-2 | Dr D Ory | N/A |
| CHO M12-AnxA6ko | This paper | N/A |
| A431-WT | ECACC | 85090402 |
| A431 AnxA6 | [[11](#_ENREF_11)] | N/A |
| MEF-WT | [[12](#_ENREF_12)] | N/A |
| MEF AnxA6ko | [[12](#_ENREF_12)] | N/A |
| COS-1 | ATCC | CRL-1650 |
|  |  |  |
| **Oligonucleotides** |  |  |
| Hamster & mouse *ORP1L* Fwd:  5’-ctggcatgctattttggaca-3’ | This paper | N/A |
| Hamster & mouse *ORP1L* Rev:  5’-agctttctttgttgagtcc-3’ | This paper | N/A |
| Hamster *Stard3* Fwd:  5’-agggtctgacaatgaatcag-3’ | This paper | N/A |
| Hamster *Stard3* Rev 5’-acagggcaggaaggtcttca-3’ | This paper | N/A |
| Hamster *Rpl13* Fwd 5’-gccccacttccacaaggatt-3’ | This paper | N/A |
| Hamster *Rpl13* Rev 5’-ataccagccaccctgagttc-3’ | This paper | N/A |
|  |  |  |
| **Chemicals and Recombinant Proteins** |  |  |
| Sandoz 58-035 | Sigma Aldrich | S9318 |
| LPDS | This paper | N/A |
| LDL | This paper | N/A |
| Puromycin | Sigma Aldrich | P8833 |
| Fetal Bovine Serum | Biological Industries | 04-001-1A |
| Trypsin | Gibco by Life Technologies | 15400-054 |
| F12 (HAM) | Biological Industries | 01-095-1A |
| DMEM | Biological Industries | 01-055-1A |
| L-Glutamine | Sigma Aldrich | 49419 |
| Penicillin-Streptomycin solution | Biological Industries | 03-031-1B |
| Lipofectamine RNAiMax | Invitrogen | 13778-075 |
| GenJet Plus Reagent | SigmaGen Laboratories | SL100499 |
| Pierce Protein A Agarose beads | Thermo Scientific | 20333 |
| Glutathione Sepharose 4B beads | GE Healthcare | 17-0756-01 |
| GenJet Plus Reagent | SignaGen Laboratories | SL100499 |
| Lipofectamine RNAiMAX | Invitrogen | 13778-075 |
| RNeasy Mini Kit | Qiagen | 74104 |
| Nitrocellulose Membranes 0.45 um | Bio-Rad | 162-0115 |
| Immobilon-P Transfer Membranes | Millipore | IPVH00010 |
| Lysing Matrix D tubes | MP Biomedicals | 6913-100 |
| Na_3_VO_4_ | Sigma-Aldrich | D6508 |
| NaF | Sigma-Aldrich | S6508 |
| PMSF | Sigma-Aldrich | P7626 |
| Aprotinin | Sigma-Aldrich | A1153 |
| Leupeptin | Sigma-Aldrich | L2884 |
| Super RX-N Fuji Medical X-Ray films | Fujifilm | 47410 19289 |
| Paraformaldehyde (PFA) | Electron Microscopy Sciences | 15710 |
| Saponin | Sigma-Aldrich | S4521 |
| BSA | Sigma-Aldrich | A7906 |
| Mowiol | Calbiochem, Merk | 475904 |
| Glutaraldehyde | Merck Millipore | 104239 |
| EZ-ECL | Biological Industries | 20-500-120 |
| Triton X-100 | Sigma-Aldrich | T8787 |
| BSA fat free | Sigma-Aldrich | A8806 |
| siMLN64-m (siStARD3) | Santa Cruz | sc-149470 |
| siAnxA6-m | Santa Cruz | sc-29689 |
| siTBC1D15-m | Santa Cruz | sc-154093 |
| siScramble | Ambion | 4635 |
|  |  |  |
| **Critical Commercial Assays** |  |  |
| Bio-Rad protein assay dye reagent concentrate | Bio-Rad | 500-0006 |
| High Capacity cDNA Reverse Transcription Kit | Applied Bioscience | 4368814 |
| PCR Brilliant SYBRGreen qPCR Master Mix | Agilent Technologies | 600828 |
|  |  |  |
| **Software and Algorithms** |  |  |
| ImageJ | [[13](#_ENREF_13)] | N/A |
| GraphPad Prism 5 | http://www.graphpad.com | N/A |
| Vesicle quantification plugin (for ImageJ) | This paper | N/A |
| CRISPR design | <https://benchling.com> | N/A |

**REFERENCES**

1. Garcia-Melero A, Reverter M, Hoque M, Meneses-Salas E, Koese M, Conway JR, Johnsen CH, Alvarez-Guaita A, Morales-Paytuvi F, Elmaghrabi YA, Pol A, Tebar F, Murray RZ, Timpson P, Enrich C, Grewal T, Rentero C (2016) Annexin A6 and Late Endosomal Cholesterol Modulate Integrin Recycling and Cell Migration. J Biol Chem 291 (3):1320-1335. doi:10.1074/jbc.M115.683557

2. Peralta ER, Martin BC, Edinger AL (2010) Differential effects of TBC1D15 and mammalian Vps39 on Rab7 activation state, lysosomal morphology, and growth factor dependence. J Biol Chem 285 (22):16814-16821. doi:10.1074/jbc.M110.111633

3. Grewal T, Heeren J, Mewawala D, Schnitgerhans T, Wendt D, Salomon G, Enrich C, Beisiegel U, Jackle S (2000) Annexin VI stimulates endocytosis and is involved in the trafficking of low density lipoprotein to the prelysosomal compartment. J Biol Chem 275 (43):33806-33813. doi:10.1074/jbc.M002662200

4. Choudhury A, Dominguez M, Puri V, Sharma DK, Narita K, Wheatley CL, Marks DL, Pagano RE (2002) Rab proteins mediate Golgi transport of caveola-internalized glycosphingolipids and correct lipid trafficking in Niemann-Pick C cells. J Clin Invest 109 (12):1541-1550. doi:10.1172/JCI15420

5. Sun Q, Westphal W, Wong KN, Tan I, Zhong Q (2010) Rubicon controls endosome maturation as a Rab7 effector. Proc Natl Acad Sci U S A 107 (45):19338-19343. doi:10.1073/pnas.1010554107

6. Itoh RE, Kurokawa K, Fujioka A, Sharma A, Mayer BJ, Matsuda M (2005) A FRET-based probe for epidermal growth factor receptor bound non-covalently to a pair of synthetic amphipathic helixes. Exp Cell Res 307 (1):142-152. doi:10.1016/j.yexcr.2005.02.026

7. Yamano K, Fogel AI, Wang C, van der Bliek AM, Youle RJ (2014) Mitochondrial Rab GAPs govern autophagosome biogenesis during mitophagy. Elife 3:e01612. doi:10.7554/eLife.01612

8. Cantalupo G, Alifano P, Roberti V, Bruni CB, Bucci C (2001) Rab-interacting lysosomal protein (RILP): the Rab7 effector required for transport to lysosomes. EMBO J 20 (4):683-693. doi:10.1093/emboj/20.4.683

9. Das A, Goldstein JL, Anderson DD, Brown MS, Radhakrishnan A (2013) Use of mutant 125I-perfringolysin O to probe transport and organization of cholesterol in membranes of animal cells. Proc Natl Acad Sci U S A 110 (26):10580-10585. doi:10.1073/pnas.1309273110

10. Ran FA, Hsu PD, Wright J, Agarwala V, Scott DA, Zhang F (2013) Genome engineering using the CRISPR-Cas9 system. Nat Protoc 8 (11):2281-2308. doi:10.1038/nprot.2013.143

11. Grewal T, Evans R, Rentero C, Tebar F, Cubells L, de Diego I, Kirchhoff MF, Hughes WE, Heeren J, Rye KA, Rinninger F, Daly RJ, Pol A, Enrich C (2005) Annexin A6 stimulates the membrane recruitment of p120GAP to modulate Ras and Raf-1 activity. Oncogene 24 (38):5809-5820. doi:10.1038/sj.onc.1208743

12. Alvarez-Guaita A, Vila de Muga S, Owen DM, Williamson D, Magenau A, Garcia-Melero A, Reverter M, Hoque M, Cairns R, Cornely R, Tebar F, Grewal T, Gaus K, Ayala-Sanmartin J, Enrich C, Rentero C (2015) Evidence for annexin A6-dependent plasma membrane remodelling of lipid domains. Br J Pharmacol 172 (7):1677-1690. doi:10.1111/bph.13022

13. Schneider CA, Rasband WS, Eliceiri KW (2012) NIH Image to ImageJ: 25 years of image analysis. Nat Methods 9 (7):671-675
