# Supplementary figures and images for: Annexin A6 modulates TBC1D15/Rab7/StARD3 axis to control endosomal cholesterol export in NPC1 cells
Source: Cell Mol Life Sci. 2019 Oct 29;77(14):2839–57. doi: 10.1007/s00018-019-03330-y (PMC7326902; doi:10.1007/s00018-019-03330-y)

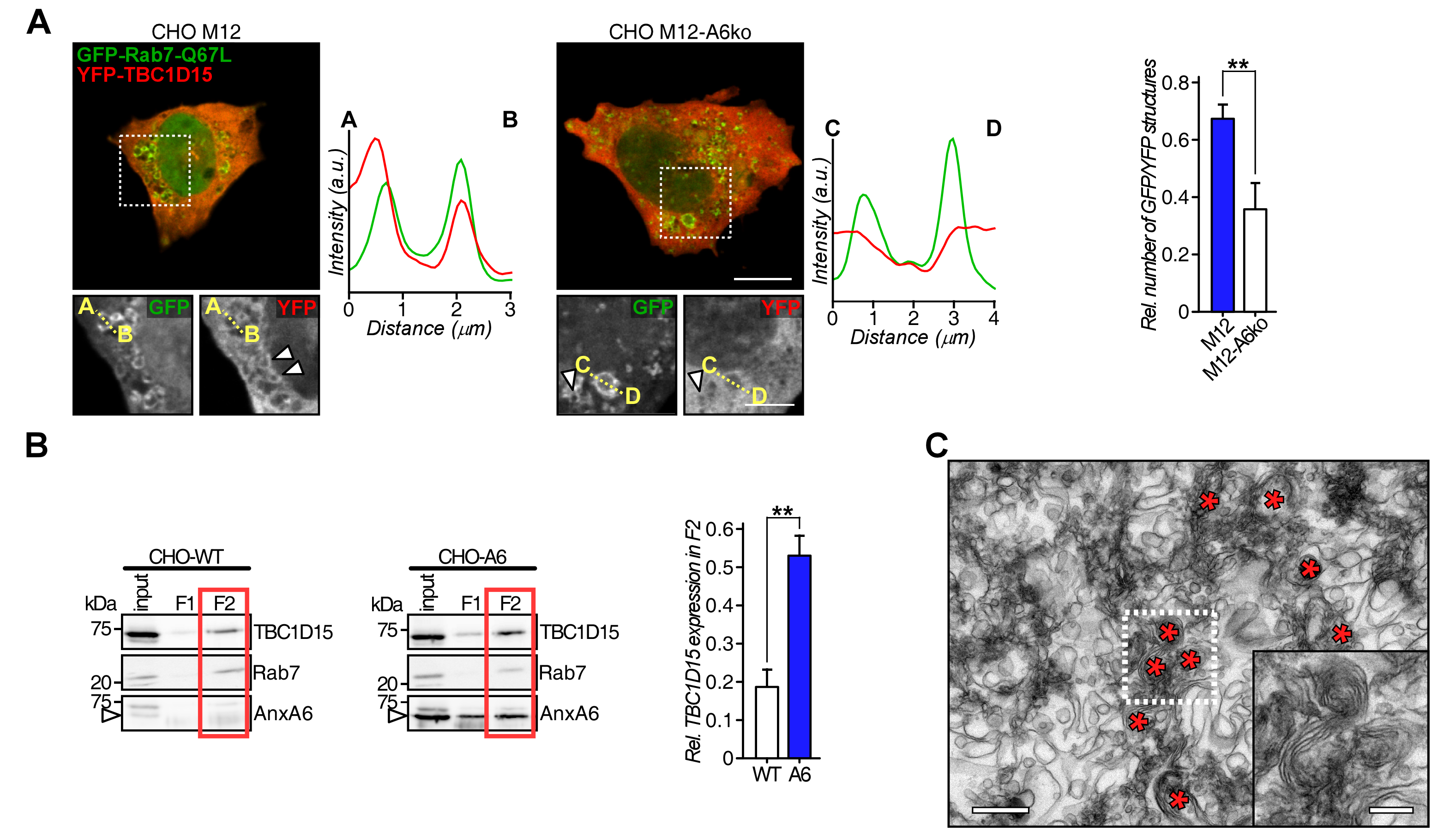

Supplement: Supplementary file 2 — Characterization of AnxA6/TBC1D15 interaction. (A) Representative images of CHO M12 and CHO M12-A6ko cells expressing GFP-Rab7-Q67L (green) and YFP-TBC1D15 (red). Areas of interest are shown at higher magnification. White arrowheads point at GFP-Rab7-Q67L positive vesicles. Line profile of fluorescence intensities of GFP-Rab7-Q67L (green) and YFP-TBC1D15 (red) are shown (A-B and C-D, 3-4 μm). Scale bar, 10 and 5 μm. Quantification shows the relative number of late endosomes with GFP/YFP (n=15 cells). (B) Subcellular fractionation of cell lysates from CHO-WT and CHO-A6 cells on discontinuous sucrose gradients. Fractions (F1-F2) were collected from top, separated by gel electrophoresis and immunoblotted for TBC1D15 and Rab7 as indicated. Cell lysates (5% of total input) are shown. Relative protein levels of TBC1D15 in the Rab7-positive late endosomal fraction (F2) were normalized to total TBC1D15 levels (see input) and are shown in the right panel (n=3). (C) Representative electron micrograph of the late endosome/lysosome (LE/Lys) fraction F2 from sucrose gradients of CHO cells (Fig. S2B). Prototypical endolysosomal structures with internal membranes can be observed (*). Insert shows a high magnification of a multilamellar structure. Scale bar: 500 and 200 nm. ** p<0.01 by two-tailed Student’s t-test (A and B). Data is shown as mean ± SEM (TIFF 2503 kb) [file 18_2019_3330_MOESM2_ESM.tiff]

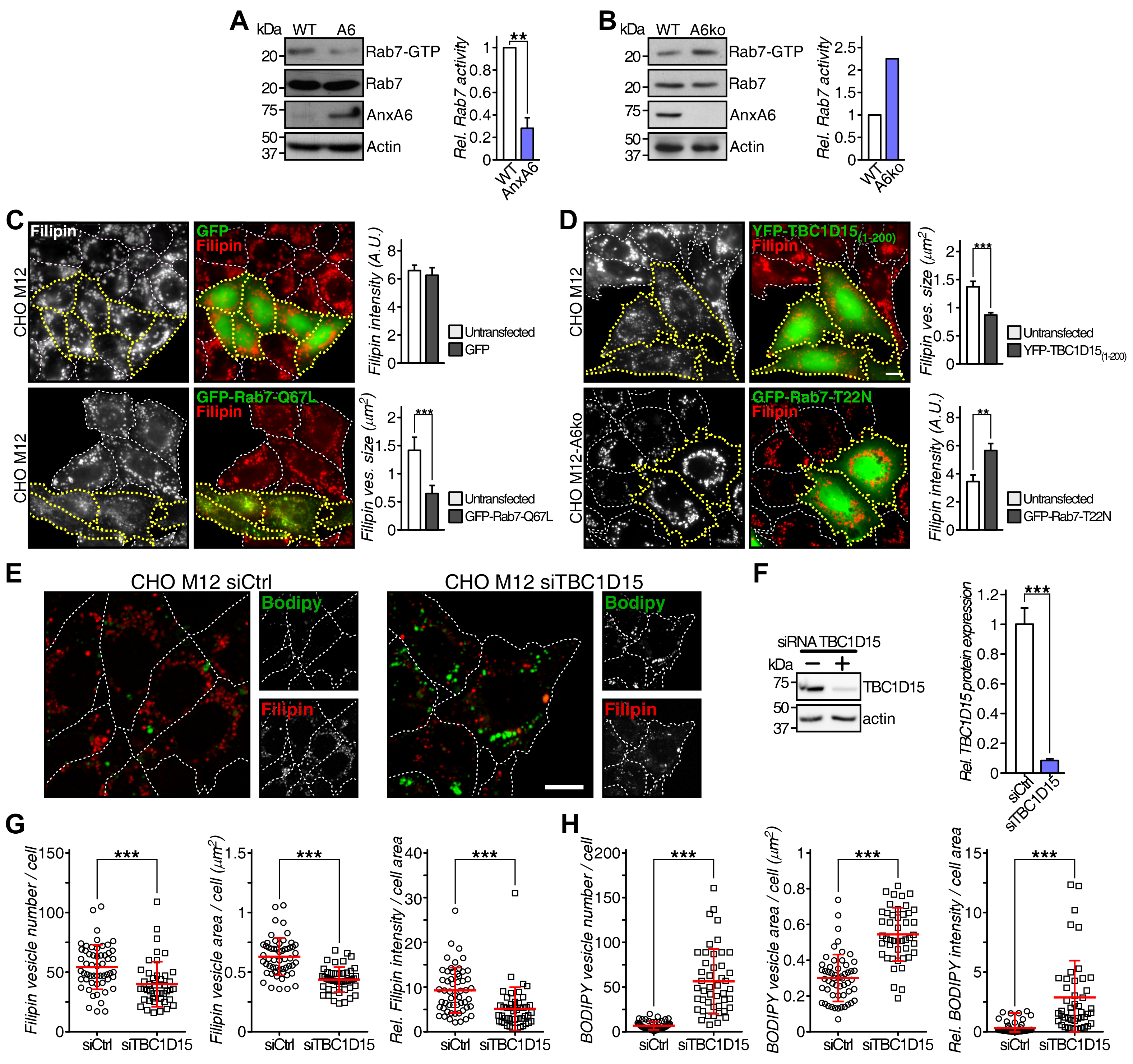

Supplement: Supplementary file 3 — Regulation of Rab7 activity and late endosome-cholesterol egress. Total levels of Rab7, AnxA6 and actin in cell lysates (5% of total input) and the quantification of relative Rab7 activity are shown (n=3). Rab7-GTP levels determined as in Fig. 2F-2H with cell lysates from (A) A431-WT and A431-A6, or (B) mouse embryonic fibroblasts from wildtype (WT) and AnxA6-KO (A6ko) mice. (C-D) CHO M12 or CHO M12-A6ko cells were transfected with empty vector (GFP), GFP-Rab7-Q67L, YFPTBC1D15( 1-200) or GFP-Rab7-T22N (green) as indicated, fixed and stained with filipin (red). For better comparison of filipin staining, the outline and shape of cells is indicated (transfected cells in yellow). Merged images are shown. Scale bar, 10 μm. The mean relative filipin intensity of at least 20 transfected vs. non-transfected cells was quantified (n=3). (E) CHO M12 cells expressing control siRNA (siCtrl) or siRNA targeting TBC1D15 (siTBC1D15) were starved in 5% LPDS for 48 h and loaded with 50 μg/ml LDL for 24 h as above. Then cells were fixed, stained with filipin (cholesterol, red) and BODIPY 493/503 (neutral lipids, green), and representative fields (merged and split channels) are shown. Enlarged regions of interest are shown. For better comparison of filipin and BODIPY staining, the outline and shape of cells is indicated. Scale bar, 10 μm. (F) Representative western blot and quantification (normalized to actin) showing siRNA-mediated TBC1D15 depletion in CHO M12 cells (n=3). (G-H) Dot-plot of number, area and relative intensity of filipin-stained (late endosomes) and BODIPY-stained (lipid droplets) vesicles per cell of a representative experiment (n > 60, 3 experiments). For quantification details see Methods. ** p<0.01; *** p<0.001 by two-tailed Student’s t-test (A, B, C, D, F, G, H). Data are presented as mean ± SEM (A, B, C, D, F) and mean ± SD in red (G, H) (TIFF 3757 kb) [file 18_2019_3330_MOESM3_ESM.tiff]

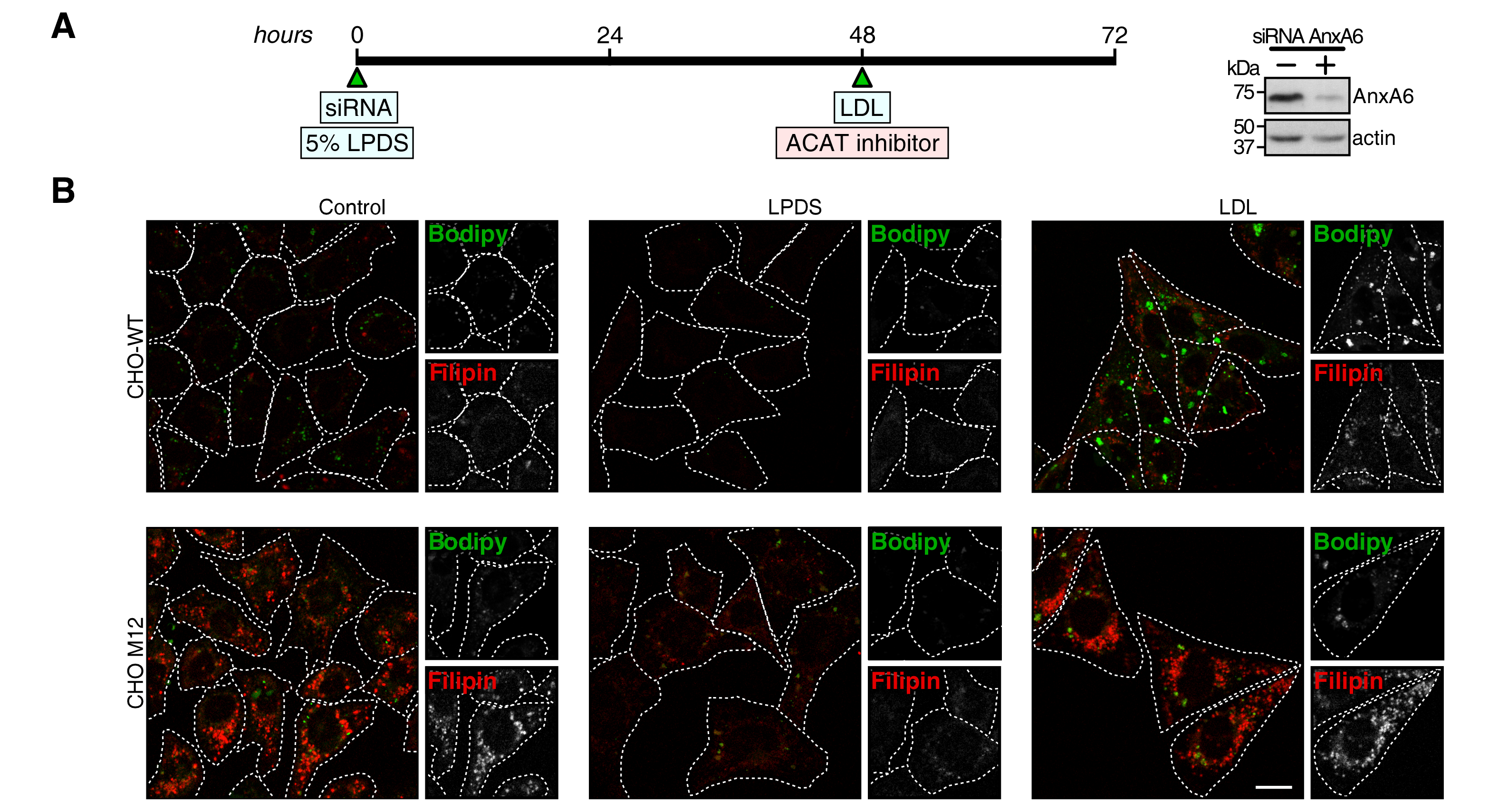

Supplement: Supplementary file 4 — Delipidation and LDL-loading experiment procedure. (A) Scheme of experimental protocol for delipidation and LDL loading, and AnxA6 siRNA depletion control in CHO M12 cells. (B) CHO-WT and CHO M12 cells were grown in 10% FCS (0 h, control), then starved in 5% LPDS for 48 h before loading with 50 μg/ml LDL for 24 h. At each time point (0, 48 and 72 h), cells were fixed, stained with filipin (cholesterol, red) and BODIPY 493/503 (neutral lipids, green). Representative fields of cells at t=0 (control), t=48 (LPDS) and t=72 h (LDL) are shown (merged and split channels). Enlarged regions of interest are shown. For better comparison of filipin and BODIPY staining, the outline and shape of cells is indicated. Scale bar, 10 μm (TIFF 2904 kb) [file 18_2019_3330_MOESM4_ESM.tiff]

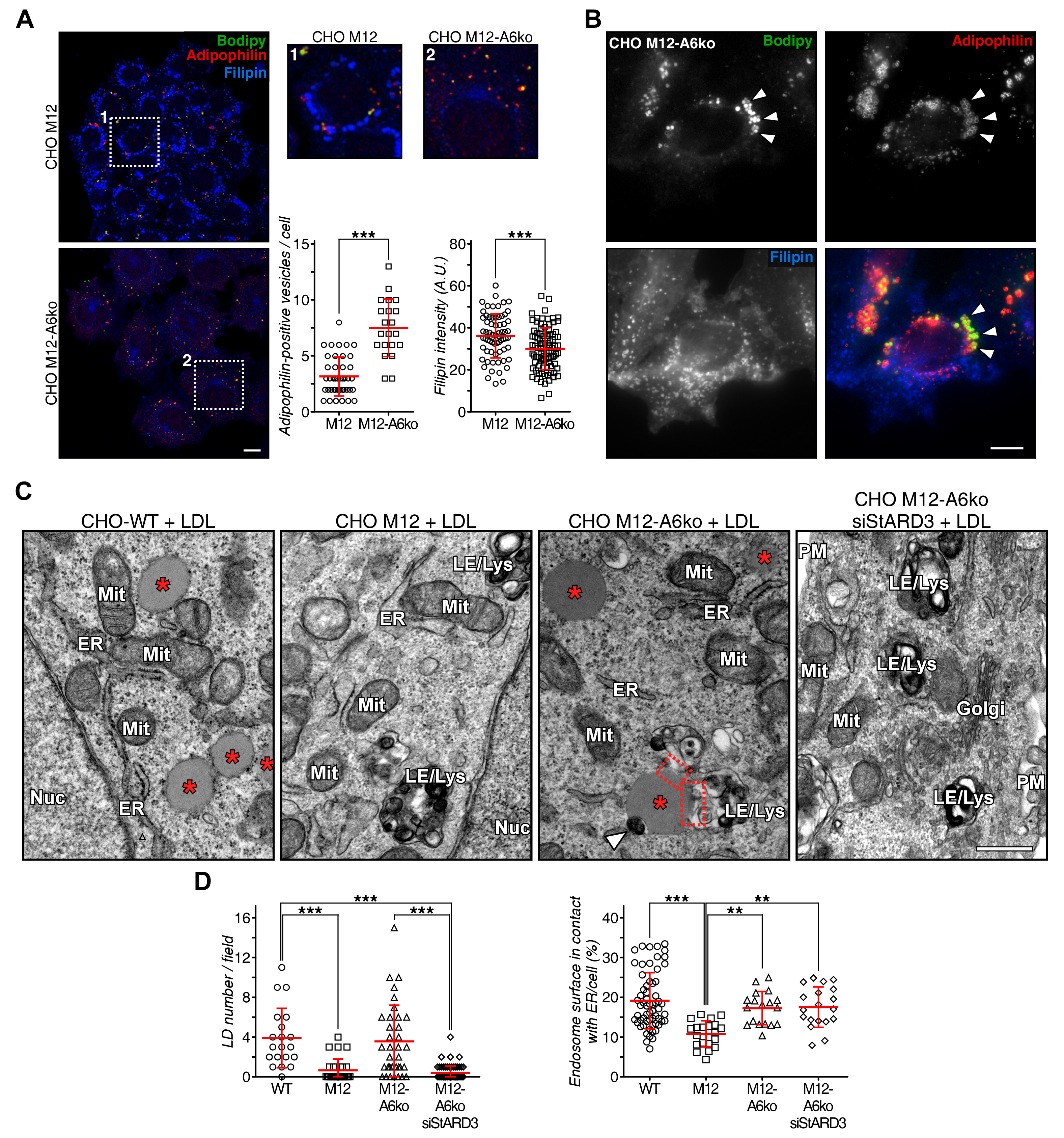

Supplement: Supplementary file 5 — Characterization of neutral lipid and cholesterol distribution in CHO M12 and CHO M12-A6ko cells. (A) CHO M12 and CHO M12-A6ko cells were grown under normal conditions. Cells were fixed, immunolabelled with the lipid droplet marker anti-adipophilin (red) and stained with filipin (blue) and BODIPY (green) as indicated. Representative images and quantification of adipophilinpositive vesicles and filipin intensity per cell (n > 20 cells, 2 experiments) are shown. For quantification details see Methods. White squares outline enlarged inserts (1-2). Scale bar, 10 μm. (B) CHO M12-A6ko cells were starved in 5% LPDS for 48 h before loading with 50 μg/ml LDL for 24 h fixed, immunolabeled with anti-adipophilin (red) and stained with filipin (blue) and BODIPY (green). Separate and merged channels are shown. Arrowheads point at representative BODIPY- and adipophilin-positive lipid droplets in the perinuclear region. Scale bar, 10 μm. (C) Conventional transmission electron microscopy (TEM) showing representative images and quantitation of lipid droplets (red asterisks) and MCS in CHO-WT, CHO M12, CHO M12-A6ko and StARD3-depleted CHO M12-A6ko (CHO M12-A6ko siRNA-StARD3) cells loaded with LDL for 24 h as indicated (see details in Methods) (D). Abundant lipid droplets, as characterized by translucent electron density, can be observed in CHO-WT and CHO M12-A6ko cells. Note the close contacts between lipid droplets and late endosomes/lysosomes (LE/Lys) structures in CHO M12-A6ko cells (red squares). Lipid droplets with an electron-dense ‘‘cap’’ (white arrow) of lipofuscinlike structures possibly pointing at early steps to initiate the sequestration/engulfing portions of LD for degradation. Mit, mitochondria; ER, endoplasmic reticulum; Nuc, nucleus; PM, plasma membrane. Scale bar, 200 nm. *** p<0.001 by two-tailed Student’s t-test (A) or one-way ANOVA with Bonferroni post-hoc test (C). All data is presented as mean ± SD in red (TIFF 8419 kb) [file 18_2019_3330_MOESM5_ESM.tiff]

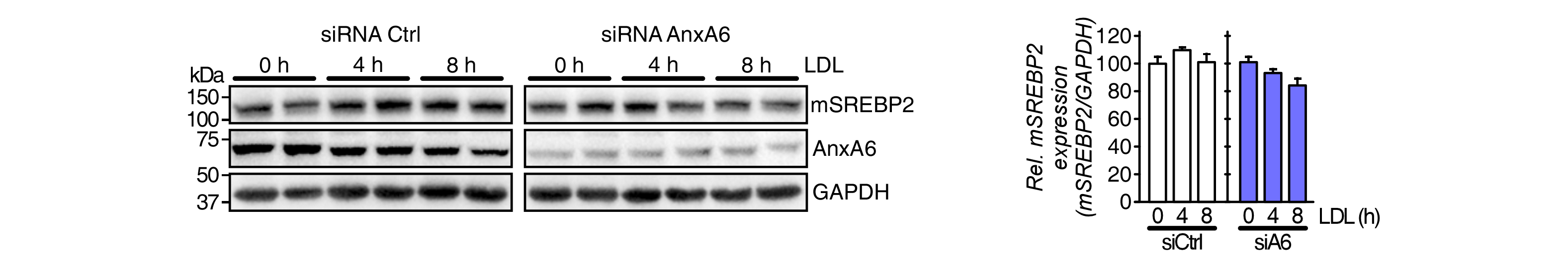

Supplement: Supplementary file 6 — Reduced expression of mature SREBP2 (mSREBP2) in AnxA6-depleted CHO M12 cells. CHO M12 expressing control siRNA (siRNA Ctrl) or siRNA targeting AnxA6 (siRNA AnxA6) were grown in 5% LPDS and 10 mM mevastatin for 3 days before loading with 50 μg/ml LDL for 0, 4 and 8 h as indicated. Whole cell lysates were prepared at each time point and analyzed by Western blotting for mature SREBP2 (mSREBP2), glyceraldehyde 3-phosphate dehydrogenase (GAPDH) and AnxA6. Relative mSREBP2 levels were quantified and normalized to GAPDH. AnxA6 depletion results in less mSREBP2 protein expression (~ 20%) after 8 h LDL loading. A representative Western blot and quantification from 2 independent experiments with duplicate samples is shown. Data is presented as mean ± SEM (TIFF 378 kb) [file 18_2019_3330_MOESM6_ESM.tiff]

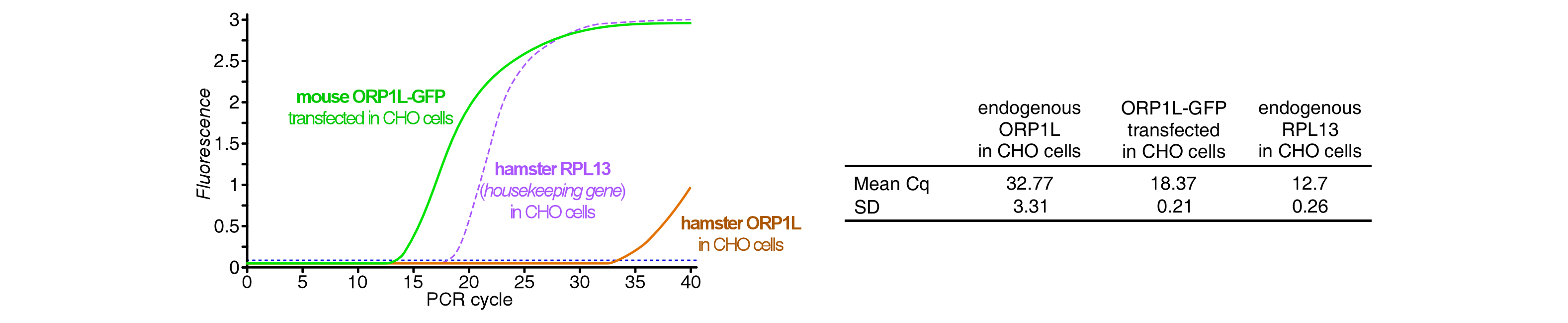

Supplement: Supplementary file 7 — Lack of ORP1L expression in CHO cells. RNA samples from CHO cells transfected ± ORP1L-GFP (mouse) were analyzed by RT-PCR for the expression of endogenous (hamster ORP1L) and transfected ORP1L-GFP (primer sequence from homologous hamster and mouse regions). The housekeeper hamster gene RPL13 served as control. While transfected ORP1L-GFP was readily detectable (mean Cq 18.37), hamster ORP1L could only be detected after more than 30 PCR cycles (mean Cq 32.77) indicating very low expression of ORP1L in CHO cells (TIFF 107 kb) [file 18_2019_3330_MOESM7_ESM.tiff]
